# Clinical and analytical validation of MI Cancer Seek^®^, a companion diagnostic whole exome and whole transcriptome sequencing-based comprehensive molecular profiling assay

**DOI:** 10.18632/oncotarget.28761

**Published:** 2025-08-13

**Authors:** Valeriy Domenyuk, Kasey Benson, Peggy Carter, Daniel Magee, Jian Zhang, Nitin Bhardwaj, Hongseok Tae, James Wacker, Foram Rathi, Siobhan Miick, Aastha Kohli, Joshua Carroll, Lori Cuyugan, Evelyn Perez, Wayeesha Zhang, John Collins, Patrick Kennedy, Jeremy Ellis, Adam Stark, Andrey Loskutov, Brittany Cuttone, Blake Taylor, Rebecca Feldman, Jeff Swenson, David Bryant, Robert Hahn-Lowry, Raunaq Kaushal, Jennifer R. Ribeiro, Jim Abraham, Milan Radovich, George W. Sledge, Matthew Oberley, David Spetzler

**Affiliations:** ^1^Caris Life Sciences, Irving, TX 75039, USA; ^*^These authors contributed equally to this work

**Keywords:** companion diagnostic, molecular profiling, solid tumors, precision oncology, next-generation sequencing

## Abstract

The precision oncology industry has evolved rapidly within the past two decades, although treatment selection remains a complex undertaking. Access to timely, accurate, and comprehensive molecular profiling data is imperative to improving patient outcomes within the expanding sphere of Food and Drug Administration (FDA)-approved targeted therapies. Caris Life Sciences has developed MI Cancer Seek^®^, an FDA-approved whole exome and whole transcriptome sequencing-based molecular test encompassing adult and pediatric tumor profiling, eight companion diagnostics (CDx), and additional laboratory developed test (LDT) capabilities. Patient tissue is maximized through simultaneous analysis of DNA and RNA with minimum input of 50 ng. The clinical and analytical validation presented herein demonstrates non-inferiority of MI Cancer Seek relative to other FDA-approved CDx tests (>97% negative and positive percent agreement), as well as its precision, sensitivity, and specificity. Accordingly, MI Cancer Seek represents a safe and effective comprehensive molecular test option supporting biomarker-directed care for oncology patients.

## INTRODUCTION

Precision oncology aims to improve patient outcomes through coupling tumor molecular profiling to specific targeted therapies. Although the number of United States Food and Drug Administration (FDA)-approved targeted therapies available to patients continues to rapidly expand, decisions regarding the use of specific therapies for select patients can be complex. Significant effort has been given to the development and application of genomic testing assays to decipher a heterogeneous molecular landscape of cancer, thus assisting clinicians in making informed therapeutic choices for patients [1–7]. The growing clinical utility of comprehensive multi-gene panels for oncology treatment planning is reflected in current recommendations by The American Society of Clinical Oncology (ASCO) [8], The European Society for Medical Oncology (ESMO) [9], and the National Comprehensive Cancer Network (NCCN) [10–14].

Sequencing of tumor DNA and RNA creates a complex portrait of the tumor genome, including identification of pathogenic variants and tumor signatures [15]. Next-generation sequencing (NGS) technologies, such as whole exome sequencing (WES), have been increasingly available in the clinical setting, while improved tumor excision and sample processing techniques have allowed for better characterization of even degraded nucleic acid found in formalin-fixed paraffin embedded (FFPE) tissues [16–18]. Whole transcriptome sequencing (WTS) can provide additional information about specific molecular alterations as well as gene expression, adding another layer of information that clinicians can use to identify appropriate treatments or clinical trials. However, techniques analyzing DNA and RNA have traditionally been performed independently [19], requiring sufficient quantities of tumor tissue to satisfy quality standards and input requirements for molecular assays.

Here, we present the clinical and analytical validation of the Molecular Intelligence (MI) Cancer Seek^®^ assay, developed at Caris Life Sciences, a College of American Pathologists (CAP)-accredited and Clinical Laboratory Improvement Amendments (CLIA)-certified laboratory. This FDA-approved (P240010) comprehensive molecular profiling assay is the evolution of the laboratory developed test (LDT) MI Tumor Seek Hybrid^®^, which has been in clinical use since February 2023. MI Cancer Seek analyzes tumor DNA and RNA simultaneously from a single total nucleic acid (TNA) extraction and targets eight companion diagnostic (CDx) indications: *PIK3CA* alterations in breast cancer; *KRAS/NRAS* wild-type status in colorectal carcinoma (CRC); *BRAF*^V600E^ mutations in CRC; *BRAF*^V600E^ or *BRAF*^V600K^ mutations in melanoma; *EGFR* exon19 deletions and L858R mutations in NSCLC; and microsatellite instability (MSI) status in endometrial carcinoma (not MSI-high; i.e., microsatellite stable (MSS)) and solid tumors (MSI-high) [20]. These indications are associated with FDA-approved targeted therapies (Table 1), based on pivotal clinical trial evidence [21–27].

**Table 1:** MI Cancer Seek CDx indications

Tumor type	Biomarker(s) and specific alteration(s) detected	Therapy
Breast (DNA only for PIK3CA)	*PIK3CA* (C420R; E542K; E545A, E545D [1635G>T only], E545G, E545K, Q546E, Q546R; and H1047L, H1047R, H1047Y)	PIQRAY^®^ (alpelisib)
Colorectal Cancer (CRC) (DNA only)	*KRAS* wild-type (absence of mutations in exons 2, 3, and 4) and *NRAS* wild-type (absence of mutations in exons 2, 3, and 4)	VECTIBIX^®^ (panitumumab)
	*BRAF* V600E	BRAFTOVI^®^ (encorafenib) in combination with ERBITUX^®^ (cetuximab)
Melanoma (DNA only)	*BRAF* V600E	*BRAF* Inhibitors approved by FDA^a^
	*BRAF* V600E or V600K	MEKINIST^®^ (trametinib) or *BRAF/MEK* Inhibitor Combinations approved by FDA^a^
Non-small Cell Lung Cancer (NSCLC) (DNA only)	*EGFR* activating mutations: *EGFR* exon 19 deletions and exon 21 (L858R) alterations	EGFR Tyrosine Kinase Inhibitors approved by FDA^a^
Solid Tumors	MSI-high	KEYTRUDA^®^ (pembrolizumab), JEMPERLI^®^ (dostarlimab-gxly)
Endometrial Carcinoma	Microsatellite stable (MSS) (Not MSI-high)	KEYTRUDA^®^ (pembrolizumab) in combination with LENVIMA^®^ (lenvatinib)

^a^For the most current information about the device indications for the therapeutic products in this group, go to: https://www.fda.gov/medical-devices/in-vitro-diagnostics/list-cleared-or-approved-companion-diagnostic-devices-in-vitro-and-imaging-tools#Group_Labeling. Abbreviations: CDx: companion diagnostic; FDA: Food and Drug Administration; MSI: microsatellite instability; MSS: microsatellite stable.

**Figure 1:**
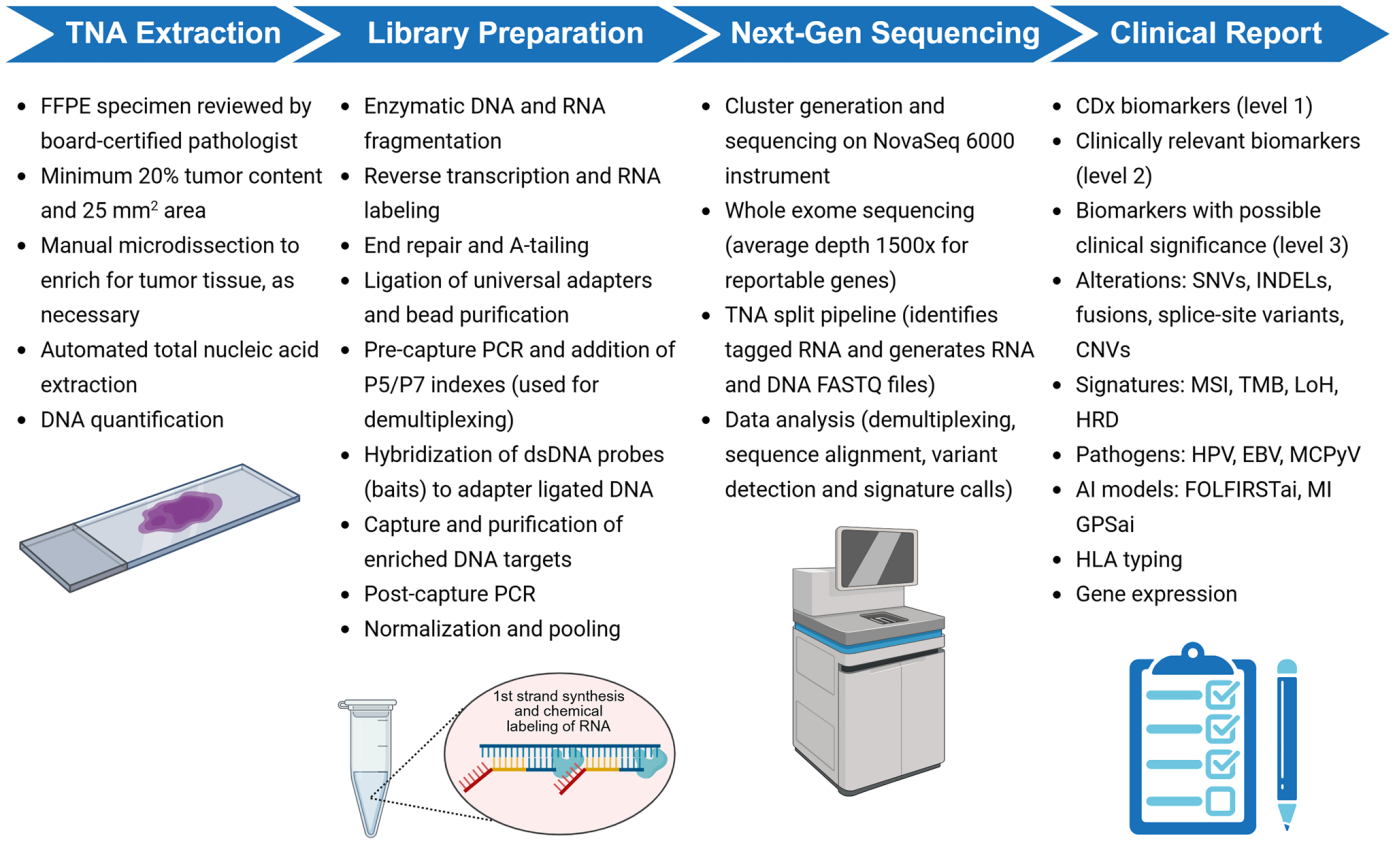
Overview of MI Cancer Seek workflow. MI Cancer Seek begins with total nucleic acid (TNA) extraction from formalin-fixed, paraffin-embedded (FFPE) tissue slides. A minimum 20% tumor content and 25 mm^2^ is required, which is obtained through manual microdissection if required. During library preparation, RNA is labeled during first strand cDNA synthesis. Sequencing is performed on qualified Illumina NovaSeq 6000 instruments. Raw data is processed by Caris’ proprietary bioinformatics pipeline, and a report is generated that includes CDx biomarkers (level 1 evidence), clinically relevant biomarkers (level 2 evidence), and biomarkers with possible clinical significance (level 3 evidence). Abbreviations: CNV: copy number variation; EBV: Epstein-Barr virus; GPS: genomic probability score; HLA: human leukocyte antigen; HPV: human papilloma virus; HRD: homologous recombination deficiency; INDEL: insertion/deletion; LoH: loss of heterozygosity; MCPyV: Merkel cell polyomavirus; MSI: microsatellite instability; SNV: single nucleotide variant; TMB: tumor mutational burden. Created in BioRender. Ribeiro, J. (2025) https://BioRender.com/m29z318.

The ‘hybrid’ methodology of MI Cancer Seek allows efficient tissue utilization and accurate and comprehensive detection of a wide range of clinically relevant biomarkers from a minimum DNA input of 50 ng isolated from microdissected, FFPE tumor tissue with minimum 20% tumor content (Figure 1). DNA is sequenced to an average depth of 230× for 20,859 genes (whole exome), 1000× for 720 genes with known and potential clinical relevance, 1500× for 228 reportable genes, and RNA to a minimum of 1.37 million total mapped reads. An annotated clinical report is generated including biomarkers that have a high-level of evidence to associate with matched FDA-approved therapies, as well as additional clinically relevant information based on published evidence. The validation presented here demonstrates that MI Cancer Seek is a robust, accurate, and precise molecular test that meets FDA standards for CDx indications and tumor mutational profiling, with additional LDT capabilities as described.

## RESULTS

### Clinical validation for FDA companion diagnostic (CDx) and tumor profiling claims

#### Non-inferiority studies for CDx claims

**Figure 2:**
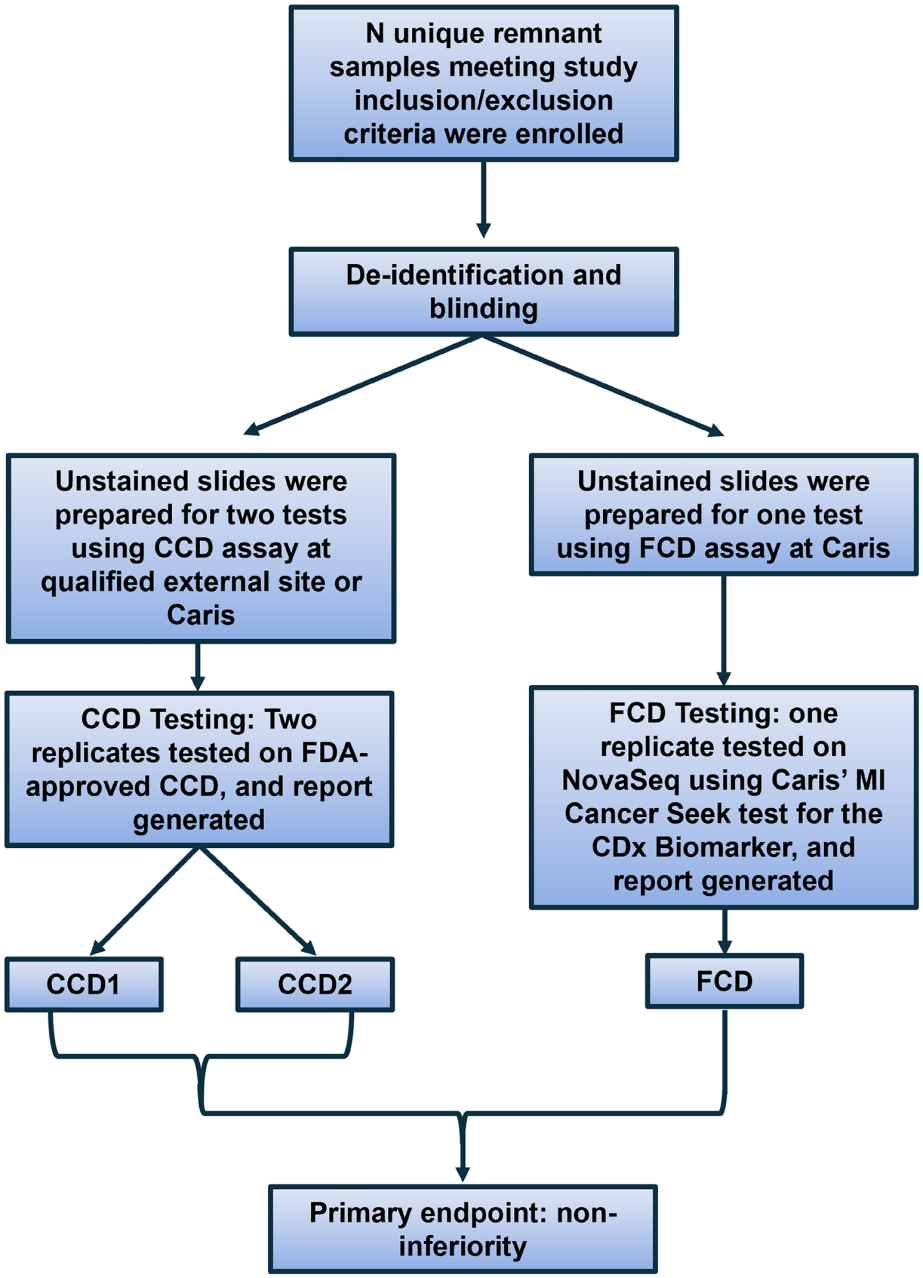
Schematic of MI Cancer Seek validation for CDx claims. Remnant samples were enrolled meeting study criteria. Samples were de-identified and lab personnel blinded to their biomarker status. Unstained slides (USS) were used to complete two tests with the comparator companion diagnostic (CCD) and one test with MI Cancer Seek as the follow-on companion diagnostic (FCD). Concordance was evaluated by determining the agreement between the two CCD replicates to the agreements of FCD to CCD1 and FCD to CCD2. Concordance with FCD was also determined using the concordance between both CCD replicates as “truth”. Non-inferiority was the primary endpoint for analysis.

MI Cancer Seek has eight CDx claims representing high clinical burden in the oncology patient population (Table 1, Supplementary Table 1). As a follow-on CDx (FCD) device, clinical validity was demonstrated through non-inferiority to an appropriate FDA-approved test (Supplementary Table 2), as described by Li et al. [28]. One replicate tested by MI Cancer Seek (FCD replicate) was compared to two replicates tested by each comparator companion diagnostic (CCD) test for each CDx claim, as shown in Figure 2. The results for FCD comparison to concordant CCD replicates are summarized in Table 2, and full concordance results are shown in Supplementary Tables 3–5. Regarding *PIK3CA* alterations in breast cancer, *KRAS/NRAS* wild-type status in advanced CRC, *BRAF*^V600E^ mutations in CRC, *BRAF*^V600E^ or *BRAF*^V600K^ mutations in melanoma, *EGFR* exon19 deletions and L858R mutations in NSCLC, and MSI status in endometrial carcinoma and solid tumors, positive percent agreement (PPA) and negative percent agreement (NPA) ranged from 97–100%, and all upper limits of the 95% confidence intervals met pre-specified non-inferiority margins, as described in ‘Methods’.

**Table 2:** Summary of PPA and NPA for MI Cancer Seek CDx clinical concordance studies

Biomarker and indication	FDA-approved Comparator Method	Sample Size, N^a^	PPA (95% CI)	NPA (95% CI)
MSI Status in Solid Tumor Types	Ventana MMR RxDx Panel	401	97.5% (94, 99.1)	98.5% (95.4, 99.7)
MSI Status in EC	Ventana MMR RxDx Panel	251	98.4% (93.8, 99.9)	97.6% (92.8, 99.5)
*BRAF*^V600E/K^ in Melanoma	bioMérieux THxID BRAF Kit	330	98.9% (95.6, 99.9)	99.3% (95.9, 100.2)
*BRAF*^V600E^ in CRC	*therascreen* BRAF V600E RGQ PCR Kit	352	99.4% (96.5, 100.2)	100% (97.3, 100.4)
*KRAS* and *NRAS* wild-type in CRC	Praxis Extended RAS Panel	262	100.0% (96.1, 100.6)	97.2% (92.7, 99.1)
*EGFR* exon 19 deletions or L858R mutations in NSCLC	Roche cobas *EGFR* Mutation Test V2	315	98.1% (91.4, 99.6)	99.4% (96.1, 100.2)
*PIK3CA* alterations in BC	*therascreen* PIK3CA RGQ PCR Kit	343	99.4% (96.4, 100.2)	100.0% (97.2, 100.4)

^a^Includes samples with complete records for analysis. There were invalid results on either CCD or FCD for *BRAF*^V600E/K^ in melanoma (*n* = 4), *BRAF*^V600E^ in CRC (*n* = 5), *KRAS* and *NRAS* wild-type in CRC (*n* = 24), *EGFR* exon 19 deletions or L858R mutations in NSCLC (*n* = 1), and *PIK3CA* alterations in BC (*n* = 5). Invalids were excluded for overall concordance results calculated using CCD1 and CCD2 consensus calls as a reference, and additional sensitivity analyses were performed to evaluate non-inferiority, including sensitivity analyses for prevalence and invalids where applicable (data not shown). Abbreviations: BC: breast carcinoma; CRC: colorectal adenocarcinoma; EC: endometrial carcinoma; MSI: microsatellite instability; NPA: negative percent agreement; NSCLC: non-small cell lung cancer; PPA: positive percent agreement.

#### Tumor profiling validation

**Figure 3:**
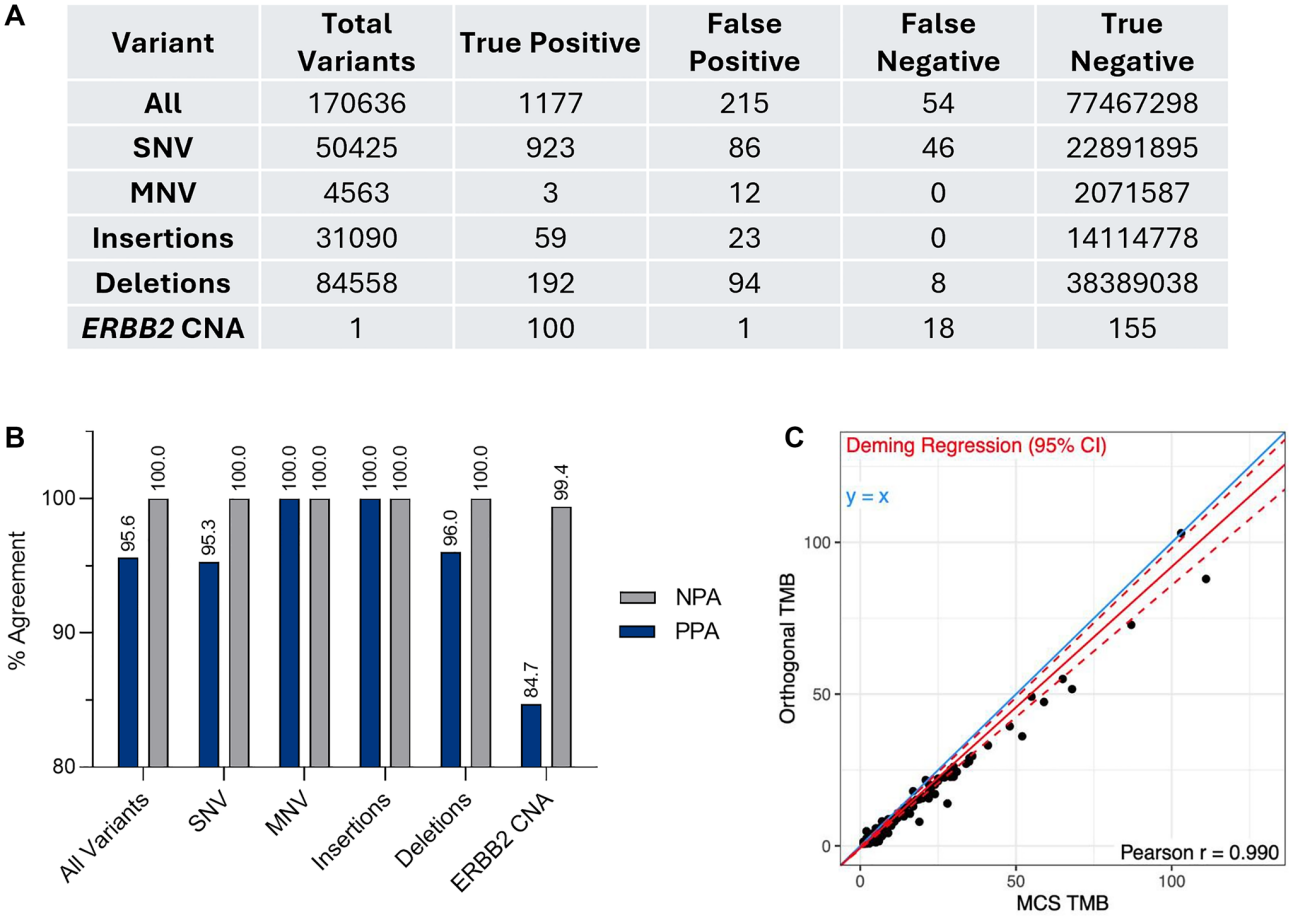
Tumor profiling validations. (**A**) Validation of variant detection by MI Cancer Seek compared to PGDx elio tissue complete and *ERBB2* copy number amplification compared to fluorescent *in situ* hybridization (FISH). Numbers of true positive (TP), true negative (TN), false positive (FP), and false negative (FN) are shown. One intermediate sample was counted as positive for *ERBB2*. One sample that was invalid by MCS and five samples that were invalid by FISH were excluded for *ERBB2*. (**B**) Positive percent agreement (PPA) and negative percent agreement (NPA) between MI Cancer Seek and the comparator assay are shown for each variant type. (**C**) Deming regression analysis of MI Cancer Seek (MCS) TMB values versus the orthogonal test values. Abbreviations: INDEL: insertion/deletion; MNV: multi nucleotide variant; SNV: single nucleotide variant; TMB: tumor mutational burden.

MI Cancer Seek provides tumor profiling for use by qualified health professionals in accordance with professional oncology guidelines. Reportable genes with boosted bait coverage are listed in Supplementary Table 6. In order to validate the detection of single nucleotide variants (SNVs) and insertions/deletions (INDELs), MI Cancer Seek results were compared to PGDx elio™ tissue complete, an FDA-cleared targeted gene panel assay [29, 30]. Among 454 eligible specimens, PPA was ≥95% and NPA was 100% for all variant types (Figure 3A, 3B). These metrics demonstrated strong concordance of MI Cancer Seek with the comparator assay. Where discordance was noted, this was determined primarily to be due to inherent differences between the two assays with regards to percent variant frequency (VF) cutoff and variant calling rules, and not due to true discordance between MI Cancer Seek and PGDx elio tissue complete. The ability of MI Cancer Seek to detect *ERBB2* (HER2) copy number amplifications (CNAs) in breast cancer was also analyzed. MI Cancer Seek results for *ERBB2* CNAs were compared to results of the PathVysion HER2 DNA Probe Kit, yielding 84.7% PPA and 99.4% NPA (Figure 3B). Due to the lower PPA for this comparison, additional clinical investigation to confirm the presence of *ERBB2* CNAs with another FDA-approved or cleared test is strongly recommended.

To validate MI Cancer Seek detection of tumor mutational burden (TMB), results from 497 eligible specimens were compared to those from a WES assay (Personalis, Inc., Menlo Park, CA, USA). Deming regression analysis demonstrated a strong correlation between MI Cancer Seek and the Personalis, Inc. WES assay (Pearson r = 0.990; slope = 0.856; intercept = −0.859) (Figure 3C). When comparing TMB using only a subset of exome regions where both assays had ≥50× depth, the concordance between MCS and the orthogonal assay improved even further (slope = 0.927; intercept = −0.68).

### Analytical sensitivity of MI Cancer Seek

**Figure 4:**
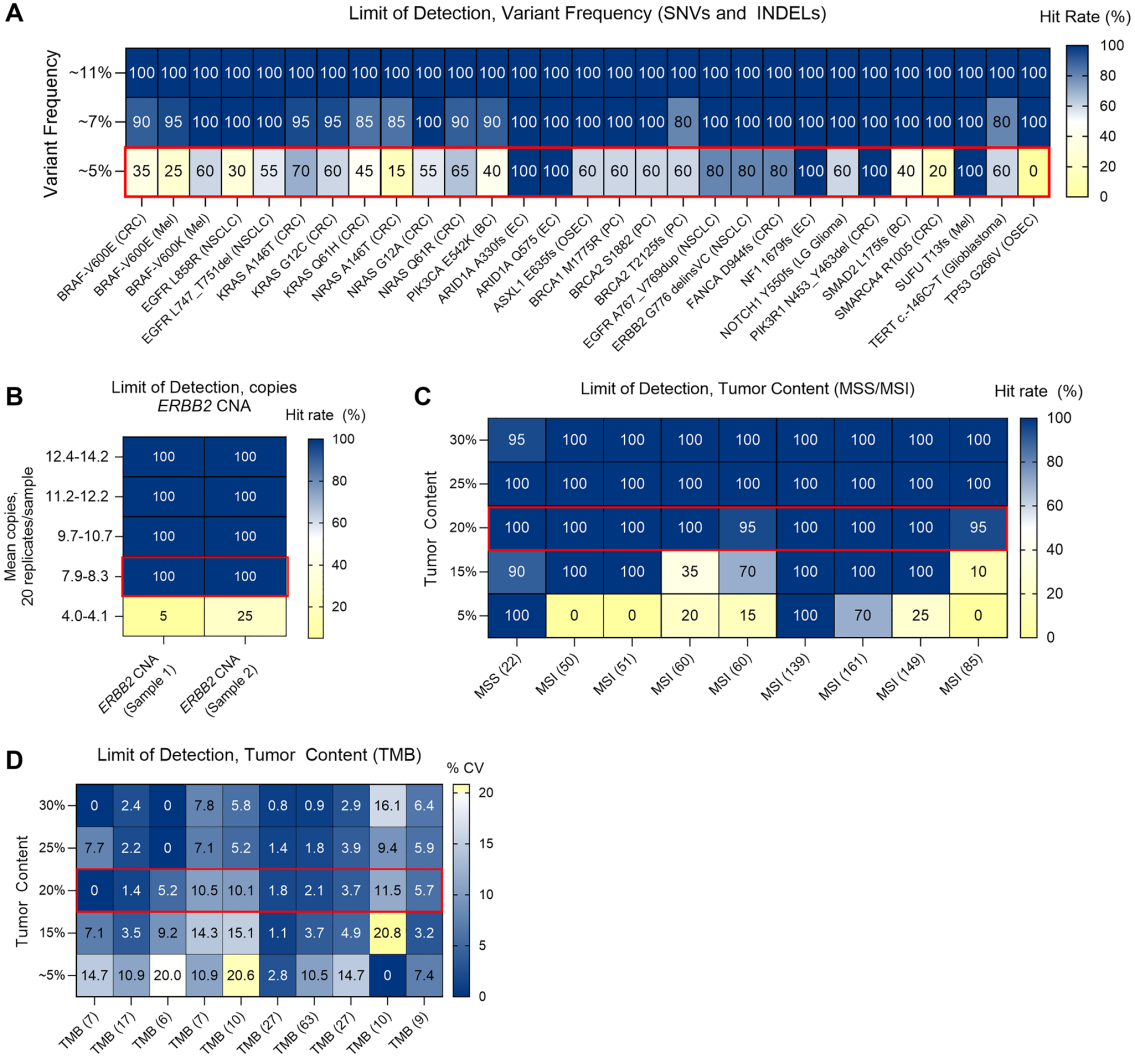
Limit of detection (LoD). (**A**) LoD for variant frequency (VF) of SNVs and INDELs (including CDx biomarkers). Caris cutoff is 5% VF for reporting. (**B**) Copies LoD for *ERBB2* CNA. (**C**, **D**) LoD for % tumor content (TC) including MSI (C), and TMB (D). Hit rate (%) at each given VF (A), nReads (B) or tumor content (C) is shown by the heatmap color, with the quantitative hit rate (%) shown in each box. The coefficient of variation (% CV) is shown for TMB in (D). The red boxes highlight the LoD cutoff employed by MI Cancer Seek. In (C, D), the quantitative values for MSI (C) and TMB (D) are indicated in parentheses. Abbreviations: BC: breast carcinoma; CNA: copy number amplification; CRC: colorectal carcinoma; EC: endometrial carcinoma; INDEL: insertion/deletion; Mel: melanoma; MSI: microsatellite instability; NSCLC: non-small cell lung cancer; OSEC: ovarian surface epithelial carcinoma; PC: prostate carcinoma; TC: tumor content; SNV: single nucleotide variant; VF: variant frequency.

Analytical sensitivity of MI Cancer Seek was evaluated through limit of detection (LoD) studies to identify the lowest VF (SNVs, INDELs) and lowest tumor content at which at least 95% of the test replicates produced a positive result. CDx biomarkers (including *BRAF*, *EGFR*, *PIK3CA*, and *RAS* mutations) and other tumor profiling biomarkers (including clinically relevant alterations and TMB) were represented in marker positive samples diluted with lineage matched marker negative samples. Observed VF was concordant with the target VF for each biomarker and reproducible across replicates (Supplementary Figure 1). The results of LoD studies support the recommended sensitivity of variant detection at >5% VF for MI Cancer Seek, illustrated by reduced detection rates at 5% VF for certain mutations (Figure 4A). LoD for *ERBB2* CNAs was determined to be 8.3 copies, which was the lowest level of copies yielding ≥95% detection (Figure 4B). Furthermore, tumor content LoD studies for MSI and TMB found reliable detection of these biomarkers at 20% cutoff (Figure 4C, 4D), which exceeded the tumor content LoD for other alterations (15% for *ERBB2* CNAs, 10.5% for all SNVs and INDELs, and 12.0% for CDx variants) (Supplementary Table 7). Thus, the 20% tumor content requirement for MI Cancer Seek is considered sufficient to enable reliable detection of all variants/biomarkers.

A limit of blank (LoB) study established the false positive rate using non-tumor wild-type (WT) samples across multiple tissue types. The results demonstrate a false positive rate of 0% for most DNA capabilities (INDELs, MSI, TMB) and <0.01% for SNVs (Supplementary Table 8). Together, there were a total of three false positive calls for TERTc.-124C>T, which were found to have VF near the variant calling threshold for MI Cancer Seek.

Finally, we performed a DNA input study to investigate the performance of MI Cancer Seek at minimum (50 ng) and optimal/maximum (220 ng) DNA input, as well as suboptimal and overloading levels (25 ng, 37.5 ng, 275 ng, and 330 ng). The PPA and NPA for SNVs, INDELs, MSI, and *ERBB2* CNAs were 100% between minimum and optimal DNA input (Table 3). At other input levels, PPA and NPA were also 100% aside from one false negative call for MSI at 37.5 ng. In addition, mean TMB was consistent between 50 ng and 220 ng: the upper bound of the 95% confidence interval (CI) for absolute percent difference in mean TMB values between control and challenge input ranged from 0.0–10.5% (Table 3 and Supplementary Figure 2).

**Table 3:** DNA input study (Concordance of 50 ng with 220 ng)

Alteration	PPA (95% CI)	NPA (95% CI)	Absolute % Difference in upper bound of CI
MSI	100 % (66.4, 100)	100 % (92.1, 100)	N/A
INDEL	100 % (73.5, 100)	100 % (100, 100)	N/A
SNV	100 % (85.8, 100)	100 % (100, 100)	N/A
*ERBB2* CNA	100% (73.5, 100)	100% (92.1, 100)	N/A
TMB	N/A	N/A	0–10.5%

Abbreviations: CI: confidence interval; CNA: copy number amplification; INDEL: insertion/deletion; MSI: microsatellite instability; SNV: single nucleotide variant; TMB: tumor mutational burden.

### Analytical specificity of MI Cancer Seek

We next evaluated analytical specificity through several studies including interfering substances (exogenous and endogenous), carry-over, and cross-contamination. Exogenous substances included paraffin, xylene, Proteinase K, and 80% ethanol, spiked at the appropriate step to mimic carryover of the substance being tested. Endogenous substances included hemoglobin, colloid, calcium/calcium phosphate, mucin, and conjugated bile acids. The impact of necrotic tissue, melanin, and fat cells on assay performance will be evaluated in a post-market study, although some of these are mitigated whenever possible through microdissection of tumor tissue. There was minimal or no effect detected for exogenous or endogenous substances on assay performance in intended tumor types (Supplementary Table 9). Likewise, carry-over (run-to-run) and cross-contamination (within run) were evaluated at the highest DNA input of 220 ng, which demonstrated 100% PPA and NPA for global mutations, SNVs, INDELs, and MSI. Further specificity studies showed that for any sample, there was a <0.004% chance of index cross-contamination (misassignment of reads to a sample due to both sample indexes being incorrectly switched to another sample). An *in silico* analysis demonstrated low off-target depth of coverage (mean 1.5×), which confirmed capture bait specificity for reportable gene regions.

### Precision of MI Cancer Seek

**Figure 5:**
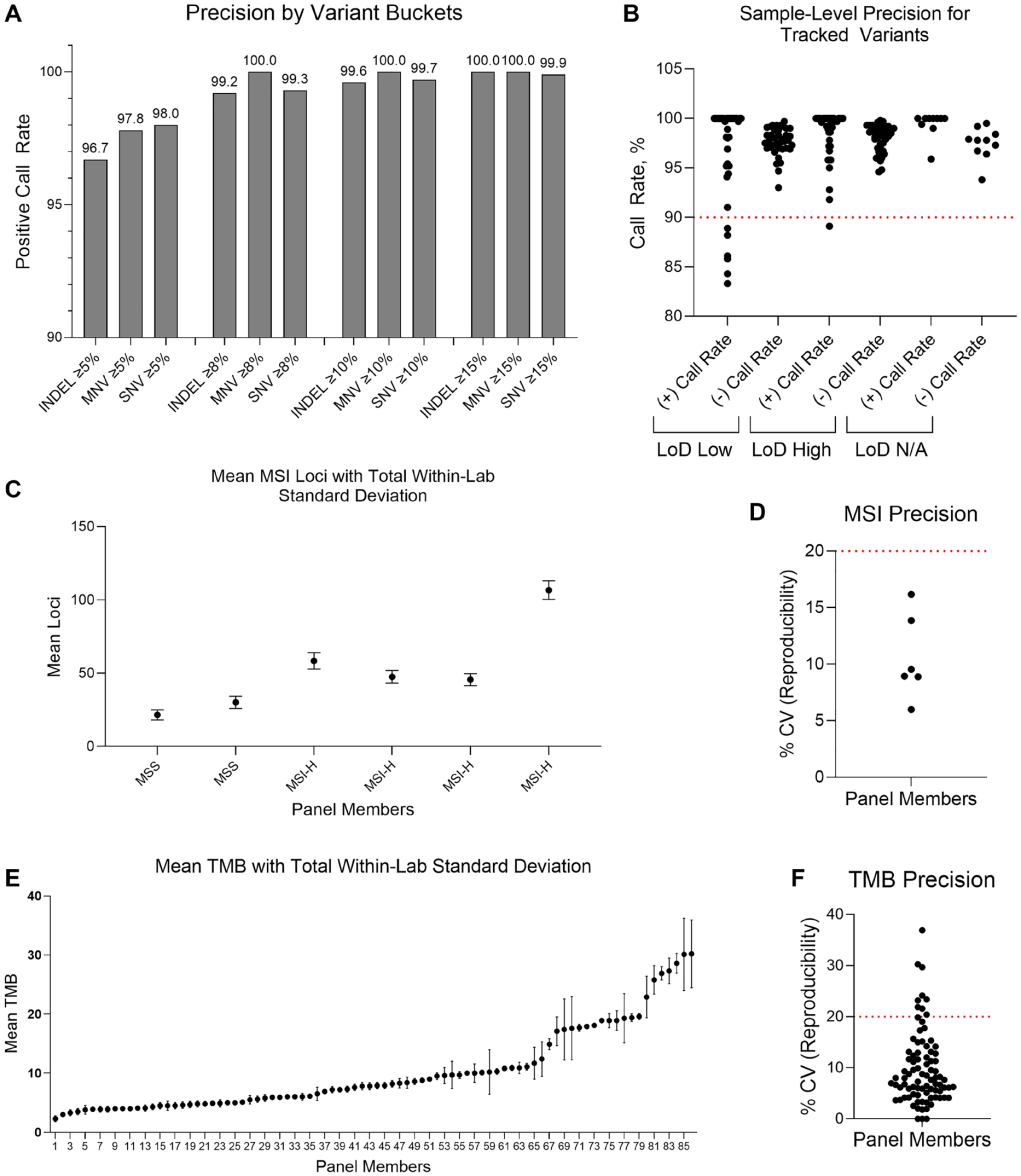
Precision study. There were 49 panel members in the precision study. Each panel member was tested a total of 36 times, including three operator teams, three instrument sets, three reagent lots, and was minimally executed across three non-consecutive days over a 20-day span. (**A**) Precision per variant frequency (VF) bucket for INDELs, MNVs, and SNVs. Variants included passenger variants that were not the basis of panel member selection. (**B**) Sample-level positive and negative calls rates for tracked variants in all 49 panel members in precision study. There were 1–19 variants per sample. 37 panel members were tested at high (2–3×) and low (1–1.5×) LoD. One additional panel member was tested only at high LoD and one additional panel member only at low LoD. The remaining panel members did not have variant frequency LoD adjusted (two panel members were MSS, four panel members were MSI-high, two panel members were TMB-high, and two panel members had an *ERBB2* CNA). (**C**) Mean MSI loci (*n* = 6) is shown with total within-lab standard deviation, including operator/instrument, reagent lot, within-run, and between-run variability. (**D**) %CV for MSI loci is shown. (**E**) Mean TMB (*n* = 49) is shown with total within-lab standard deviation, including operator/instrument, reagent lot, within-run, and between-run variability. TMB was measured in all 86 samples (including panel members tested at high and low LoD). (**F**) %CV for TMB is shown. For (D) and (F), the red dotted line indicates acceptable %CV. Abbreviations: CNA: copy number amplification; CV: coefficient of variation; INDEL: insertion/deletion; LoD: limit of detection; MNV: multi-nucleotide variant; MSI-H: microsatellite instability high; MSS: microsatellite stable; SNV: single nucleotide variant; TMB: tumor mutational burden.

Next, we performed a precision analysis at low (1–1.5×) and high (2–3×) LoD levels to evaluate the total within-lab variability expected in the MI Cancer Seek assay by evaluating both the individual and combined impact of reagent lots, instruments, operators, and non-consecutive run days across a panel of samples containing representative types of genetic alterations detected by the test. For SNVs, multi-nucleotide variants (MNVs), and INDELs, precision increased according to variant buckets (≥5%, ≥8%, ≥10%, and ≥15% VF), but exceeded 96% even at the lowest VF bucket (Figure 5A). Sample level precision analysis also revealed overall high positive and negative call rates at high and low LoD, although some samples had reduced positive call rates at low LoD (Figure 5B). All CDx markers achieved 100% agreement across replicates except for three cases that had borderline MSI status resulting in agreements of 97.2%, 91.7%, and 94.4%. Coefficient of variation (CV), which is the ratio of the standard deviation to the mean and is a measure of assay precision, was determined for MSS/MSI-high (measured by quantitative loci) and TMB (measured across a range of values from 2.3–30.2), incorporating operator/instrument, reagent lot, within-run, and between-run variability (Figure 5C–5F). Together, the results of these analyses support the precision of MI Cancer Seek for tumor profiling and CDx biomarker detection at LoD thresholds.

### Laboratory developed test (LDT) validation

MI Cancer Seek is the FDA-approved version of its LDT predecessor, MI Tumor Seek Hybrid, which provides additional information relevant to indicated tumor types. While findings other than those listed in Table 1 for MI Cancer Seek are not prescriptive or conclusive for labeled use of any specific therapeutic product, these capabilities have been validated internally and meet CAP/CLIA requirements. Therefore, they may be considered at the physician’s discretion when evaluating possible treatments or clinical trials, or for research purposes. These LDT capabilities include the following: copy number variants (CNVs; including 1p19q co-deletion and 7+/10- co-occurrence), karyotype images, structural variants (SVs)/fusions, splice site variants (SSVs; including *MET* exon 14 skipping, *AR*v7, *EGFR*vIII), human leukocyte antigen (HLA) typing, loss of heterozygosity (LoH), homologous recombination deficiency (HRD) for predicting PARP inhibitor response in ovarian cancer, FOLFIRSTai signature for predicting first-line chemotherapy response in metastatic CRC, Genomic Probability Score (GPSai) for predicting tissue of origin in cancers of unknown primary (CUP), cancer-associated virus detection (HPV, EBV, MCPyV), and gene expression. Several of these capabilities have been or will be described in detail elsewhere, including HRD, FOLFIRSTai, and GPSai. The validation of HRD in ovarian cancer has been fully described [31]. Given the evolving landscape and potential clinical applications of this marker, HRD will also be reported for research-use-only on all tumor types, with further validations forthcoming. Development of the FOLFIRSTai 67-gene signature and the original GPSai model are described by Abraham et al. [32–34]. The GPSai model has since been updated and clinically validated, which has been reported separately. When compared to validated internal or external comparator assays (Supplementary Table 10), MI Tumor Seek Hybrid fulfilled acceptance criteria with regards to accuracy and precision for all LDT capabilities (Table 4).

**Table 4:** Concordance and precision of MI Tumor Seek Hybrid with comparator assays

LDT Capability	PPA (95% CI) accurate/total	NPA (95% CI) accurate/total	Precision (Average consensus call rate)
CNA^a^	100% (93.7, 100) 57/57	99.9% (99.8, 100) 29,435/29,437	99.3%
CNV loss^b^	94.4% (86.6, 97.8) 68/72	98.9% (98.7, 99.0) 15,760/15,941	94.1%
*ERBB2* (HER2) CNA	92.1% (89.2, 94.4) 409/444	99.6% (99.3, 99.8) 3,510/3,524	N/A
1p19q co-deletion	100% (78.5, 100) 14/14	100% (85.7, 100) 23/23	N/A
7+/10- co-occurrence	100% (78.5, 100) 14/14	96.2% (81.1, 99.3) 25/26	N/A
SV/fusion, SSV^c^	98.2% (90.4, 99.7) 54/55	N/A^d^	SV/fusion: 93.0%
*ALK* fusion	94.3% (90.8, 96.8) 249/264	99.8% (99.7, 99.9) 8,932/8,949	N/A
HLA-A (other than A*2:01)	99.3% (97.5, 99.8) 280/282	N/A	100%
HLA-A*2:01	99.5% (97.3, 99.9) 201/202	N/A	100%
HLA-B	98.6% (97.0, 99.3) 477/484	N/A	100%
HLA-C	97.9% (96.2, 98.9) 474/484	N/A	100%
LoH	91.8% (82.2, 96.4) 56/61	92.3% (87.8, 95.2) 191/207	90.7%
HRD (MI Exome)	100% (70.1, 100) 9/9	100% (64.6, 100) 7/7	92.5%
HRD (MyChoice)	100% (51.0, 100) 4/4	100% (51.0, 100) 4/4	
FOLFIRSTai	92.6% (76.6, 97.9) 25/27	92.9% (68.5, 98.7) 13/14	N/A
HPV^e^	94.0% (83.8, 97.9) 47/50	99.4% (97.7, 99.8) 308/310	100%
EBV	100% (87, 100) 26/26	100% (87, 100) 26/26	
MCPYV	100% (92.7, 100) 49/49	100% (84.5, 100) 21/21	

^a^Data shown for 325 reportable genes. ^b^Data shown for 112 reportable genes. ^c^ *MET* exon 14 skipping, *AR*v7, *EGFR*vIII. ^d^There were only two negative specimens according to MI Transcriptome, which were detected as positive by MI Tumor Seek Hybrid. These two false positive observations were determined to be from samples with 1 fusion read, which is below threshold in the comparator assay (MI Transcriptome) but exceeds the threshold of 75 nReads in MI Tumor Seek Hybrid. ^e^PPA, NPA, and OPA were ≥90% for each HPV subtype (16, 18, 31, 33, 45). Abbreviations: CNA: copy number amplification; CNV: copy number variation; EBV: Epstein-Barr virus; HLA: human leukocyte antigen; HPV: Human papilloma virus; HRD: homologous recombination deficiency; LDT: laboratory developed test; LoH: loss of heterozygosity; MCPYV: Merkel cell polyomavirus; NPA: negative percent agreement; PPA: positive percent agreement; SSV: splice site variant; SV: structural variant.

Separate validations were performed in 3,968 samples for *ERBB2* amplification in breast cancer as a complement to the MI Cancer Seek tumor profiling validation, and in 9,213 samples for *ALK* fusions in NSCLC. For this LDT validation, *ERBB2* CNA results were compared to results of Ventana’s PATHWAY anti-HER2/neu (4B5) IHC test. These large-scale validations focusing on these clinically relevant alterations [35–38] in their respective tumor types yielded PPAs of 92.1% and 94.3% and NPAs of 99.6% and 99.8%, respectively (Table 4). An additional real-world analysis of MSI status in a cohort of 46,976 solid tumors and 3,488 endometrial tumors also demonstrated high concordance with the Ventana MMR RxDx Panel (Supplementary Table 11), further supporting our CDx claim.

Finally, MI Tumor Seek Hybrid provides gene expression data for panels of genes that are determined on a lineage basis for matching to clinical trials. Transcripts per million (TPM) and the accompanying percentile in the Caris cohort of the tumor type profiled are included in the report. Validation of MI Tumor Seek Hybrid gene expression data required a different approach due to the continuous nature of the data as well as technical differences from the comparator WTS assay, MI Transcriptome. We first determined area under the receiver operating characteristic curve (AUROC) for key genes that could be validated against immunohistochemistry (IHC). MI Tumor Seek Hybrid and MI Transcriptome demonstrated comparable AUROC values relative to pathologist-signed IHC results for androgen receptor (AR), HER2, estrogen receptor (ER), progesterone receptor (PR), and Claudin-18 (AUROC >0.89 for all) (Supplementary Figure 3). We then compared TPM between MI Transcriptome and MI Tumor Seek Hybrid numerically by applying a percentile transformation method, which is described in Supplementary Figure 4. Using this method, there was a strong correlation between MI Transcriptome and MI Tumor Seek Hybrid for a set of frequently reported genes (*n* = 214) (Supplementary Figure 5). Quantitative gene expression reporting by MI Tumor Seek Hybrid was also determined to be sufficiently precise, especially at TPM ≥25 (Supplementary Figure 6). Together, the gene expression validation studies support this LDT capability and culminate the comprehensive molecular reporting of MI Cancer Seek/MI Tumor Seek Hybrid.

## DISCUSSION

We developed MI Cancer Seek (CDx) as a unique molecular assay designed to analyze both RNA and DNA simultaneously with a minimal DNA input of 50 ng and enhanced sequencing depth of genes recurrently altered in cancer. The technological workflow of MI Cancer Seek promotes efficient tissue utilization while providing comprehensive information on clinically actionable molecular vulnerabilities and additional clinical insights to be interpreted at the physician’s discretion. The results reported herein establish the clinical concordance of the MI Cancer Seek assay with appropriate comparator assays, including several FDA-approved tests. The CDx and tumor profiling claims for MI Cancer Seek are supplemented with relevant LDT capabilities including HRD, cancer-associated virus detection, and AI modeling. Together, the accuracy analysis substantially achieved or exceeded the acceptance criteria set forth, supporting MI Cancer Seek’s non-inferiority claims and CDx status.

Broad-based biomarker panels for precision medicine improve outcomes in a simplified, cost-effective manner [39–42] and are now recognized by major clinical oncology organizations such as ASCO as affording many advantages over more limited molecular testing [8]. Over the past several years, there has been an ever-expanding repertoire of newly FDA-approved targeted therapies or additional clinical indications for previously approved therapies with molecular biomarkers defining eligibility [8, 43–45], such as the tissue-agnostic approval of pembrolizumab for patients with MSI-high or TMB-high unresectable or metastatic solid tumors [46, 47]. Clinical trials testing various multi-modal therapy approaches including combinations of molecularly targeted therapies, immunotherapies, and chemotherapeutic agents further expand a physician’s biomarker-directed armamentarium [8, 48]. The importance of timely and accurate multi-gene testing to help guide these treatment decisions is underscored by data showing that that a large percentage of patients has at least one potentially actionable alteration [49, 50], while an absence of actionable alterations can help avoid unnecessary treatments that come with additional costs and side effects [51]. Multi-gene panel testing may also uncover reportable pathogenic variants in tumor tissue from patients not meeting criteria for hereditary genetic testing or may prompt eligibility for further germline testing [52–54].

Notwithstanding the advantages of multi-gene panels for oncology testing, multiple barriers exist that prevent their widespread clinical adoption, including lack of physician awareness and insufficient reimbursement for tests [43, 55]. Furthermore, inefficiencies in CDx testing such as poor test performance and poor sample quality or quantity may lead to significant loss in the oncology market and potentially severe repercussions for patients precluded from biomarker-matched therapy [43, 56]. Bearing these challenges in mind, integration of CDx tests into broad-based, multi-gene panels that optimize limited tissue resources is an essential step toward connecting as many patients as possible with personalized therapies [8].

Beyond the molecular test itself, a well-annotated and informative clinical report is essential for helping clinicians connect patients with molecularly-matched, FDA-approved, off-label, or trial therapies with varying levels of clinical consensus on the strength of the biomarker. A summary of reportable ranges and information included in the MI Cancer Seek clinical report is shown in Supplementary Table 12. Caris Life Sciences substantially adheres to and expands upon the joint consensus recommendations from the Association for Molecular Pathology, American College of Medical Genetics and Genomics (ACMG), ASCO, and CAP for essential elements of genomic test reports [57], including a tiered system for reporting the clinical significance of detected variants. Annotations are provided for each biomarker, with associated interpretive comments including functional, prognostic, predictive, and mechanistic data available from the literature. To enhance clarity and physician comprehension, cancer-type relevant biomarkers and associated therapies are highlighted, with all pathogenic and likely pathogenic variants subsequently reported. Whenever a relevant test fails due to issues with the sample (e.g., inadequate sample quantity or quality) or sequencing (e.g., insufficient depth), a notation is included in the report. While specific clinical trial recommendations are not made, a list of available clinical trials relevant to the identified biomarker is provided on the report. Clinicians also have access to the Clinical Trials Connector within their MI Portal interface, allowing them to perform real-time clinical trial searches based on location, biomarker, drugs, and trial sponsor. For appropriate patient groups, updates are provided in the form of a letter to the treating physician based on recent FDA drug approvals, further enhancing the potential impact of testing.

Collectively, the data in this validation indicate that the MI Cancer Seek assay is accurate, precise, and displays high analytical sensitivity and specificity. One limitation to be considered is the low PPA for *ERBB2* CNA detection. While we demonstrated higher PPA for the comparison of *ERBB2* CNA and HER2 IHC in our large LDT cohort (92.1%), the PPA for *ERBB2* CNA versus FISH was suboptimal (84.7%). This difference in PPA may be the result of some discordance between FISH and IHC, which is known to occur particularly in weakly positive cases [58]. Thus, we suspect that MI Cancer Seek may identify cases with higher HER2 levels with greater accuracy than intermediate cases. Additionally, results from LDT capabilities of MI Tumor Seek Hybrid and any associated therapy recommendations are not to be taken as prescriptive or conclusive. Nonetheless, this assay demonstrates equivalency with orthogonal clinically validated tests and commercial, FDA-approved molecular tests, with the added advantage of simultaneous RNA and DNA analysis from the same minimal tissue input. In addition to providing an efficient molecular test option for patients/providers, the comprehensive molecular data produced by MI Cancer Seek/MI Tumor Seek Hybrid may also contribute to the compendium of tumor molecular information for research purposes. All components of the assay were validated by Caris under CAP/CLIA regulations for high-complexity laboratory testing, and it therefore represents an FDA-approved [20], efficient biomarker panel test that will contribute to the continued improvement and accessibility of precision medicine in oncology practice.

## MATERIALS AND METHODS

### General methodology for validation

All samples, kits, reagents, and supplies were pre-specified and acquired in advance of the validation plan execution. All operators were pre-identified and trained on the workflow. All operators, samples, kit lot numbers, reagent lot numbers, expiration dates, instrument serial numbers, run dates, and validation results were documented. For any failures, inconsistencies, and/or questions, the technical supervisor (a board-certified molecular geneticist), and/or a board-certified pathologist was consulted. The studies were executed over multiple runs, by multiple operators and with different lots of reagents to ensure that testing performance was reproducible across multiple variables. The majority of cases for accuracy studies used an optimal input of DNA (220 ng), unless otherwise indicated. Non-optimal inputs (25, 37.5, 50, 275, and 330 ng) were tested for a DNA input and guard banding study. A summary of the study designs and parameters for all clinical and analytical studies performed is shown in Supplementary Table 13. Samples for the accuracy analysis underwent orthogonal testing using other Caris in-house assays or were tested using commercial assays by qualified laboratories (Supplementary Tables 2, 10).

### Samples

A total of 2,063 unique samples, representative of 48 tumor lineages, were enrolled in the clinical validation studies. For clinical validation studies, biomarker positive samples were enrolled consecutively starting from the most recent case moving backward, and an equal number of date-matched biomarker negative samples were enrolled. Samples for the *KRAS/NRAS* study were enrolled consecutively regardless of biomarker status, since this study did not require biomarker enrichment. Samples were excluded from testing if they did not meet minimum requirements for MI Cancer Seek or the comparator assay. Samples with invalid results on MI Cancer Seek or the comparator assays were excluded from the primary analysis but were analyzed in best- and worst-case sensitivity analyses. For clinical validation studies, blinding was performed so that no internal or external operator was aware of the biomarker status of the tested sample. The demographics of the cohorts (age and sex) were comparable to the cohorts from the pivotal clinical trials investigating each biomarker. Samples for analytical validation studies shown here (e.g., DNA input, LoD, precision, interfering substances, LoB, cross-contamination, carry-over) were selected based on sample biomarker status and where applicable were diluted with normal tissue samples or other cancer samples as indicated to achieve designated DNA input levels or tumor purity, VF, etc. according to prespecified study designs. Additional samples were used for the tumor profiling validation, analytical validation, and LDT validation studies (Supplementary Table 13). Positive and negative controls specific to each capability of the assay were included in this study and are included for each MI Cancer Seek run.

### Total nucleic acid (TNA) extraction

FFPE slides underwent review by a board-certified pathologist to measure tumor content. For accuracy analyses, a minimum of 20% tumor content across 25 mm^2^ area was required, with manual microdissection performed to meet requirements if necessary. Microdissection was also performed as needed to exclude interfering substances as much as possible (melanin, adipose, necrosis). TNA was auto-extracted using a MagMax FFPE DNA/RNA Ultra extraction kit (Thermo Fisher Scientific, Waltham, MA, USA) on a Biomek i7 Automated Workstation (Beckman Coulter, Brea, CA, USA) and KingFisher Flex (Thermo Fisher Scientific). DNA quantification was performed using a Quant-iT 1X dsDNA High Sensitivity Assay Kit (Thermo Fisher Scientific) and read on a GloMax^®^ Microplate Reader (Promega, Madison, WI, USA). Samples were prepared for quantification, normalized, and plated for downstream processes using a Biomek i7 Automated Workstation. RNA quantification was not required, since starting DNA and RNA molecules were converted into libraries for NGS in the same hybrid workflow. The RNA/DNA ratio in TNA from tissue was always >1; thus, the input into library preparation was controlled by limiting material (DNA).

### MI Cancer Seek library preparation and next-generation sequencing

Library preparation was automated on the Bravo B Liquid Handler (Agilent, Santa Clara, CA, USA) using KAPA NGS Library preparation reagents (Roche Diagnostics, Indianapolis, IN, USA) and custom cDNA primers (IDT, Coralville, IA, USA; GeneLink, Elmsford, NY, USA). NGS library preparation is a fully customized workflow, referred to as a ‘hybrid’ workflow, where starting TNA molecules proceed through the process together. DNA and RNA underwent fragmentation, followed by cDNA synthesis from RNA, end repair and A-tailing of cDNA and original DNA, ligation of universal adapters, pre-capture PCR, and addition of P5/P7 indexes. Genomic targets were enriched through hybridization with a custom bait panel and captured using 120 nucleotides long, double-stranded, biotinylated baits. The bait panel was customized to add additional coverage for clinically relevant and reportable genes (Supplementary Table 6). Capture and purification of enriched DNA targets was performed following hybridization of dsDNA probes (baits) to adapter-ligated DNA. Post-capture PCR was performed on enriched DNA targets, and the resulting library was quantified using a Quant-iT 1X dsDNA High Sensitivity Assay Kit. Finally, normalization and pooling were performed, with quantification of the final library pool by a Qubit 1X dsDNA High Sensitivity Assay Kit (Thermo Fisher Scientific). Sequencing was performed on a NovaSeq 6000 instrument (Illumina, San Diego, CA, USA), using recommended reagents. A Veriti Thermocycler (Thermo Fisher Scientific) was used for all PCR steps. Quality control elements were checked at applicable steps. RNA was chemically labeled during library preparation, which enables separating RNA-originated data during bioinformatics data analysis. The MI Cancer Seek workflow is shown in Figure 1.

### MI Cancer Seek bioinformatic pipeline

Sequencing data were extracted into split FASTQ files (RNA and DNA) for further processing through Caris’ proprietary bioinformatics pipeline. For RNA, Spliced Transcripts Alignment to a Reference (STAR) software was used for alignment, and STAR-Fusion was employed for fusion detection [59]. Transcripts per million (TPM) molecules were generated using the Salmon expression pipeline [60]. DNA variants detected were mapped to reference genome hg38, and well-established bioinformatics tools such as BWA, Samtools, Pindel, and snpEff were incorporated to perform variant calling functions; germline variants were filtered with various germline databases, including dbSNP. CNVs were detected using CNVkit [61]. Genetic variants identified were interpreted by board-certified molecular geneticists and annotated as ‘pathogenic,’ ‘likely pathogenic,’ ‘variant of unknown significance,’ ‘likely benign,’ or ‘benign,’ according to ACMG standards.

### Tumor signatures

The threshold to determine MSI-high status was 39 or more loci with frameshift mutations out of 5,721 loci examined. TMB was measured by counting all non-synonymous missense, nonsense, in-frame insertion/deletion, and frameshift mutations found per tumor that had not been previously described as germline alterations in dbSNP151, Genome Aggregation Database (gnomAD, AC>0) databases, or benign variants identified by Caris geneticists. A cutoff point of ≥10 mutations per MB was used based on the KEYNOTE-158 pembrolizumab trial [62].

LoH was determined by calculating the LoH of single nucleotide polymorphisms (SNPs) within 552 chromosome segments of 2–6 Mb in length. 250K SNPs were analyzed, with a minimum of 17 SNPs per Mb of genome sequence. The final call of genomic LoH was based on the percentage of all 552 segments with observed LoH (High ≥16%; Equivocal ≥11% and ≤15%; Low <11%; Indeterminate ≤3,000 SNPs read or sample depth <200×). A normal epithelial ovarian genome (NA12878) that has no non-polymorphic variants, gene fusions or other cancer hallmarks, was used as a negative control. LoH was also used to determine HRD. The presence of LoH and large-scale state transitions (LST), which are the loss of chromosomal segments larger than 10 Mb, contributed to the genomic scar score (GSS). HRD was called when the sample was positive for *BRCA1* or *BRCA2* mutation or was genomic scar score (GSS)-high (GSS ≥46), and negative when it did not meet those criteria. HRD was restricted to ovarian cancer cases.

### Statistical analysis

Sample sizes for CDx studies were targeted to achieve at least 80% power to rule out non-inferiority, accounting for projected invalid rates based on historical data. The agreement between two replicates of the comparator companion diagnostic (CCD1 and CCD2) was compared to the agreements of the follow-on companion diagnostic (FCD) to CCD1 and FCD to CCD2, according to Li et al. where a reference standard is not available and the concordance study sample is not a random sample from the MI Cancer Seek intended use population (with the exception of *KRAS/NRAS*, since no enrichment was required) [28]. Concordance results were used to calculate agreement values (NPA and PPA) and differences (𝜁) in those values. Differences in agreements were calculated according to the following formulas: 𝜁PPA1 = PPA _C1C2_ − PPA_C1F_; 𝜁PPA2 = PPA _C2C1_ − PPA_C2F_; 𝜁NPA1 = NPA _C1C2_ − NPA_C1F_; and 𝜁NPA2 = NPA _C2C1_ − NPA_C2F_. NPA/PPA_C1C2_ is the proportion of CCD1 negative/positive results in which CCD2 is negative/positive. NPA/PPA_C1F_ is the proportion of CCD1 negative/positive results in which FCD is negative/positive. NPA/PPA_C2C1_ is the proportion of CCD2 negative/positive results in which CCD1 is negative/positive. NPA/PPA_C2F_ is the proportion of CCD2 negative/positive results in which FCD is negative/positive. Prevalence-adjusted agreement values (i.e. adjusted for enrichment of the sample population) were calculated using Bayes’ Theorem and the Law of Total Probability.

FCD was considered non-inferior to CCD by a margin of ε, where ε is the maximum upper limit of the 95% CIs for each 𝜁, and δ is the target non-inferiority margin. ε1 is the true non-inferiority margin for FCD as a predictive test, ε0 is the true non-inferiority margin for FCD as a selective test, δ1 is the target non-inferiority margin for FCD as a predictive test, and δ0 is the target non-inferiority margin for FCD as a selective test. If ε <δ, FCD achieved the targeted non-inferiority level. Two-sided 95% confidence intervals (CIs) for agreement differences (𝜁) were calculated using the percentile bootstrap method with 1000 bootstrap samples for all studies except *KRAS/NRAS*, which used the continuity-corrected Wilson interval for paired data, as described in method 10 of Newcombe et al. [63]. Sensitivity analyses were performed to evaluate the impact of prevalence using an upper and lower bound based on values reported in the literature. Standard approaches in JMP Version 17 or higher, SAS Version 9.4 or higher, or R version 4.3.0 or higher were used for data analysis.

## SUPPLEMENTARY MATERIALS



## Abbreviations

ACMG: American College of Medical Genetics and Genomics
AR: androgen receptor
ASCO: American Society of Clinical Oncology
AUROC: area under the receiver operating characteristic curve
CAP: College of American Pathologists
CCD: comparator companion diagnostic
CDx: companion diagnostic
CI: confidence interval
CLIA: Clinical Laboratory Improvement Amendments
CNA: copy number amplification
CNV: copy number variation
CRC: colorectal cancer
CUP: cancer of unknown primary
ER: estrogen receptor
ESMO: European Society for Medical Oncology
FCD: follow-on companion diagnostic
FDA: Food and Drug Administration
FFPE: formalin-fixed paraffin-embedded
GPS: genomic probability score
GSS: genomic scar score
IHC: immunohistochemistry
INDEL: insertion/deletion
LDT: laboratory developed test
LoB: limit of blank
LoD: limit of detection
LST: large-scale state transitions
MI: Molecular Intelligence
MSI: microsatellite instability
MSS: microsatellite stable
NCCN: National Comprehensive Cancer Network
NGS: next-generation sequencing
NPA: negative percent agreement
NSCLC: non-small cell lung cancer
PPA: positive percent agreement
PR: progesterone receptor
SNV: single-nucleotide polymorphism
TMB: tumor mutational burden
TNA: total nucleic acid
TPM: transcripts per million
VF: variant frequency
WES: whole exome sequencing
WT: wild-type
WTS: whole transcriptome sequencing
