# Supplementary material for: Clinical and analytical validation of MI Cancer Seek®, a companion diagnostic whole exome and whole transcriptome sequencing-based comprehensive molecular profiling assay
Source: Oncotarget. 2025 Aug 13;16:642–59. doi: 10.18632/oncotarget.28761 (PMC12581394; doi:10.18632/oncotarget.28761)
Supplement: Supplementary file 1 [file oncotarget-16-28761-s001.pdf]

Clinical and analytical validation of MI Cancer Seek®, a companion diagnostic whole exome and whole transcriptome sequencing-based comprehensive molecular profiling assay

SUPPLEMENTARY MATERIALS

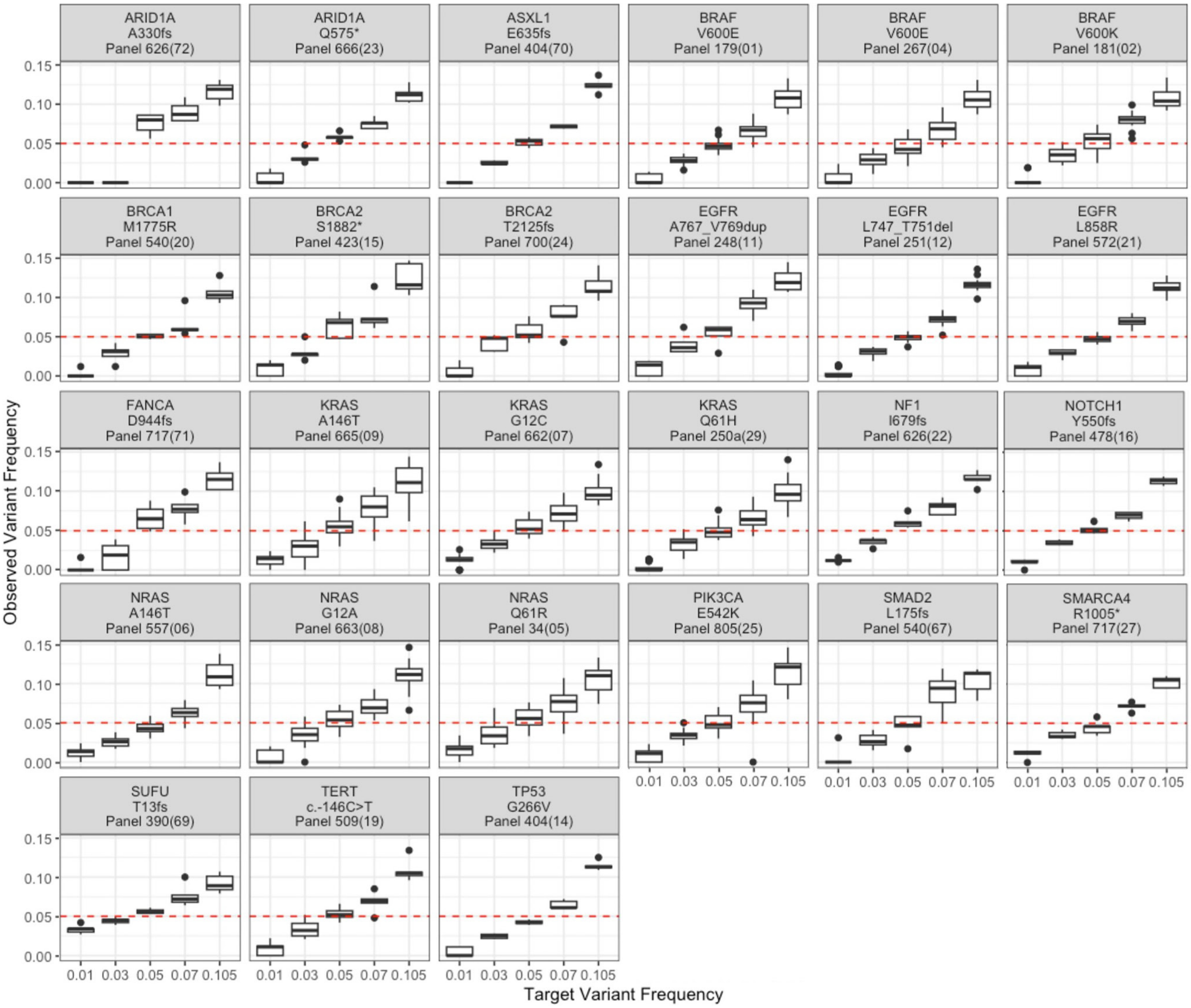

Supplementary Figure 1: Comparison of observed variant frequency with targeted variant frequency of SNVs and INDELs. Targeted variant frequencies (VF) (1%, 3%, 5%, 7%, 11%) plotted on the x-axis were achieved by diluting with marker negative samples. Observed VFs for each sample replicate for each biomarker are plotted on the y-axis. The black midline represents the median.

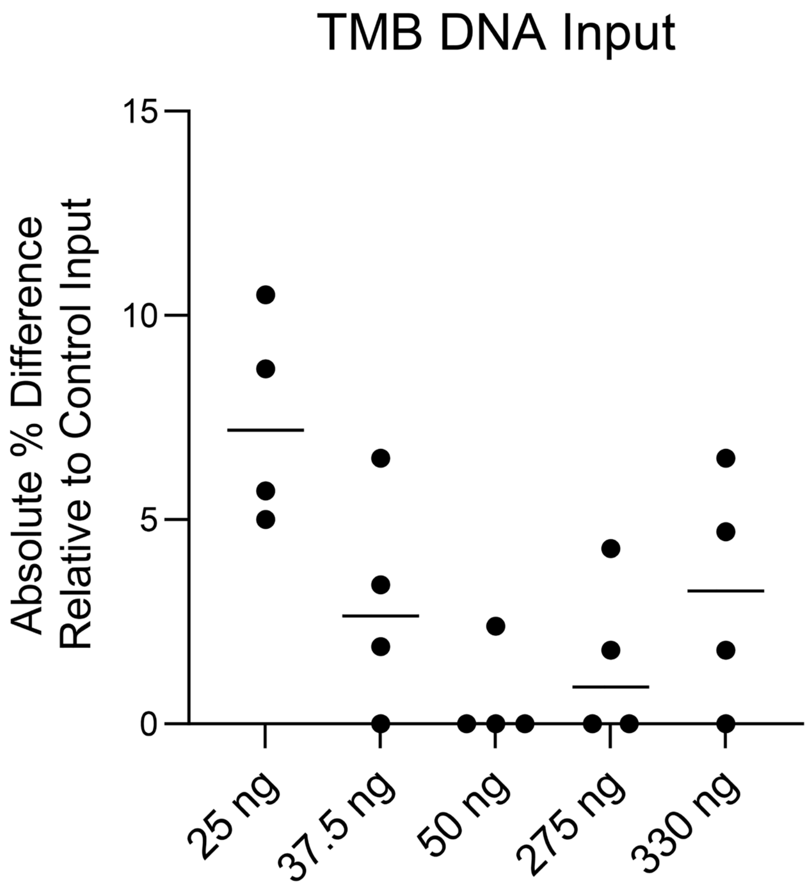

Supplementary Figure 2: DNA input for tumor mutational burden (TMB). Individual data points are shown for the absolute percent difference of TMB values at designated DNA input amounts relative to control input. Control input for 25 ng and 37.5 ng was 50 ng; control input for 50 ng, 275 ng, and 330 ng was 220 ng. Midline represents median.

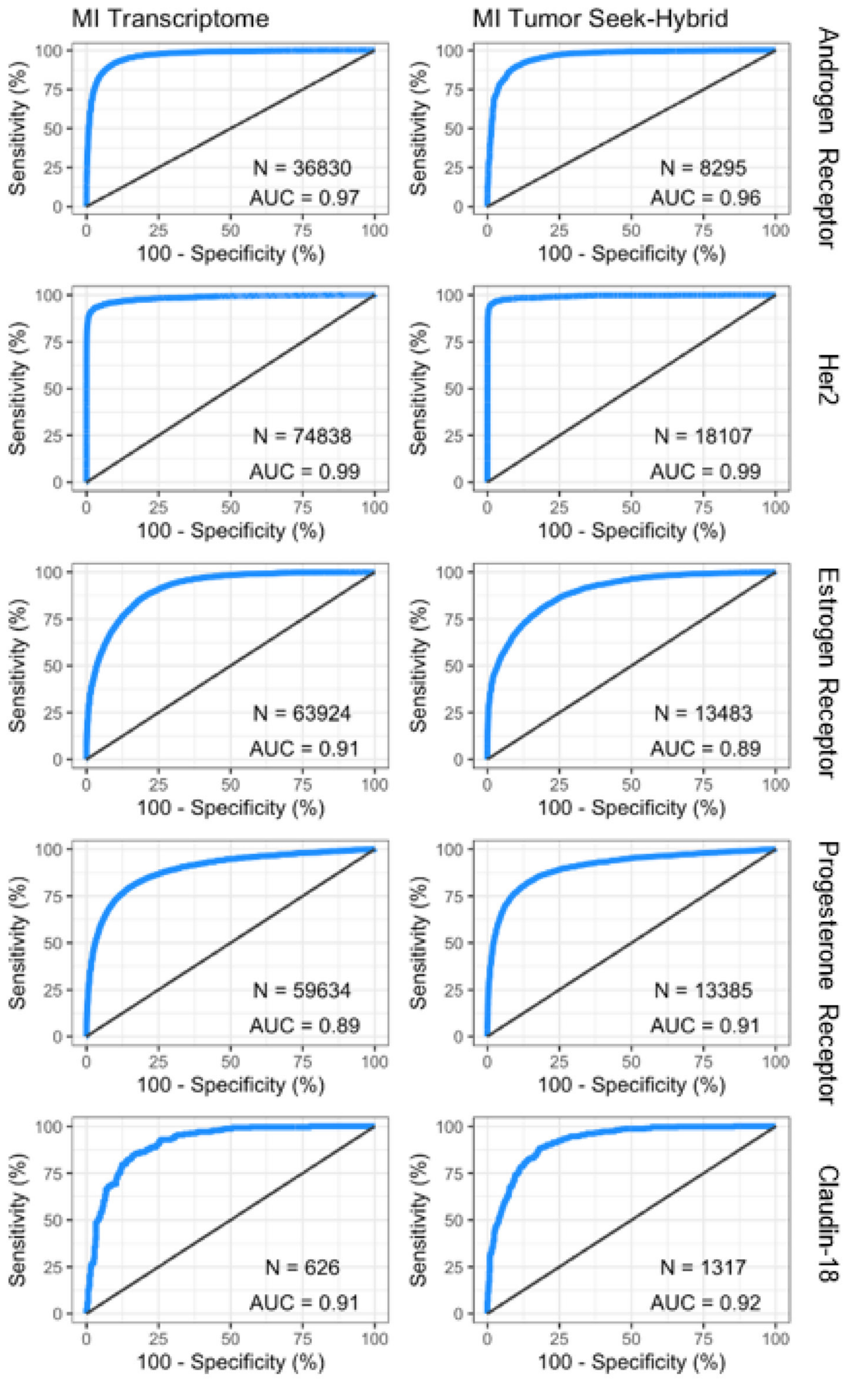

Supplementary Figure 3: MI Tumor Seek Hybrid gene expression validation. Receiving operator characteristics (ROC) curves for comparison of androgen receptor (AR), HER2, estrogen receptor (ER), progesterone receptor (PR), and Claudin-18 immunohistochemistry (IHC) to MI Transcriptome (left panels) and MI Tumor Seek Hybrid (right panels). Abbreviation: AUC: area under the curve.

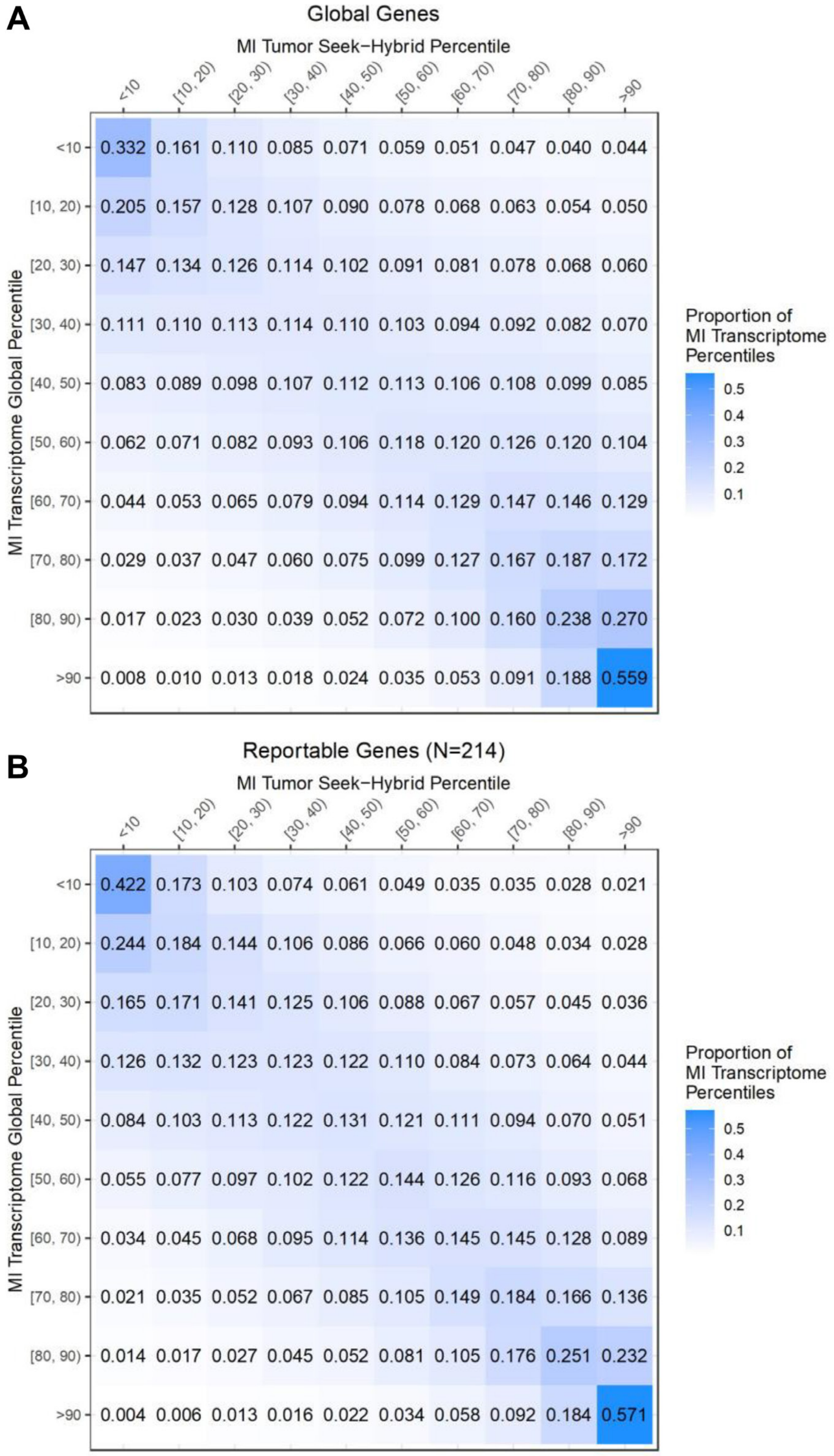

Supplementary Figure 4: Validity of percentile transformation method. To perform percentile transformation, TPMs from one assay were transformed to TPMs of the opposite assay by matching the percentile expression from one assay to the other. (**A**, **B**) Heatmap of deciles observed via the MI Transcriptome vs MI Tumor Seek Hybrid assays among all genes (global, A) and reportable genes (B). Each row sums to 1.0, representing the proportion of observed Hybrid percentiles across all genes and cases for a given percentile observed in the original MI Transcriptome run. The most heavily shaded region being the diagonal from left to right suggests a high degree of "percentile matching" between MI Transcriptome and MI Tumor Seek Hybrid.

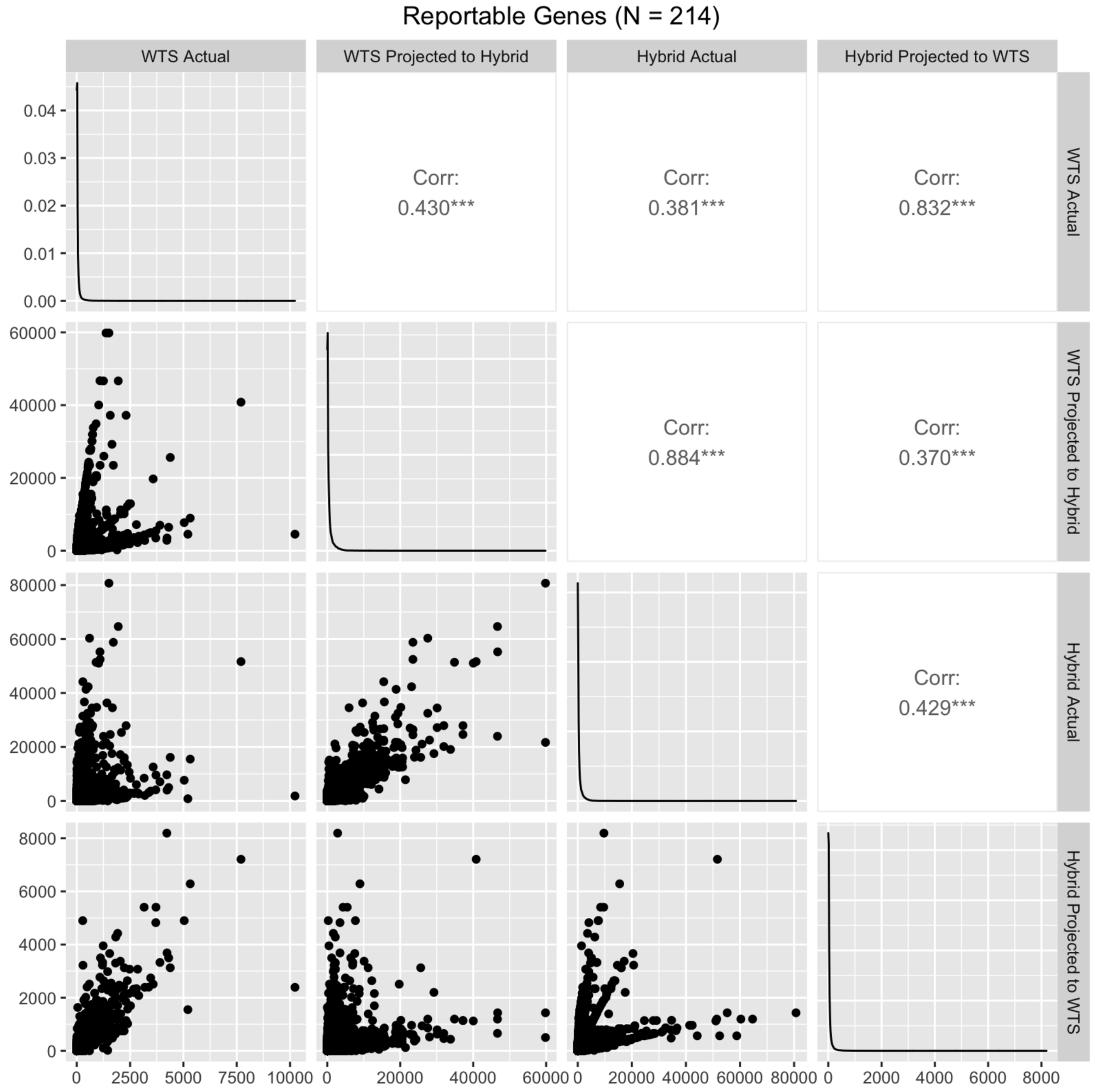

Supplementary Figure 5: Comparison of percentile-transformed MI Transcriptome TPM to MI Tumor Seek Hybrid TPM for reportable genes. Pairwise scatterplots of raw expression data (WTS Actual, Hybrid Actual) and percentile-transformed expression data (WTS Projected to Hybrid, Hybrid Projected to WTS). The upper section of the diagonal represents the Pearson correlation coefficient for the transverse cell in the bottom left corner. For example, the Pearson r between MI Transcriptome data projected to MI Tumor Seek-Hybrid like values is 0.884 (third column, second row) and the corresponding scatterplot is in the second column, third row. Raw expression values feature a mixture of well-correlated genes as well as uncorrelated genes, leading to a v-shaped pattern. Comparatively speaking, the Pearson correlation coefficients nearly double when the percentile transformations are applied.

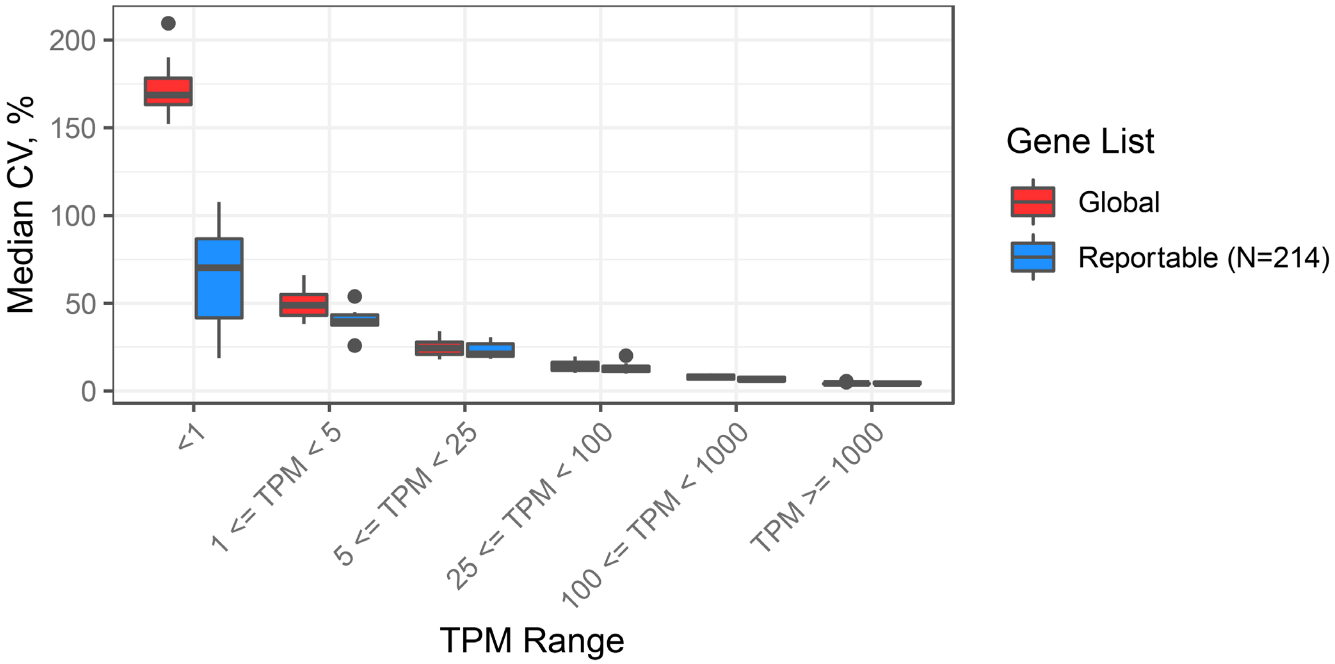

Supplementary Figure 6: Precision analysis for gene expression data. Coefficient of variation (CV) was calculated for each TPM bucket for all genes (global) and reportable genes ($N = 214$). It is important to consider that since the CV is a function of both the mean value and standard deviation (CV = σ/μ), the lower mean values will necessarily produce higher CV values with a fixed standard deviation relative to larger mean values. For example, when the standard deviation is 1.0 TPM, the CV when the mean TPM is 2.0 is 50%, however if the mean TPM is 10 the CV becomes just 10%. Thus, a conventionally attractive CV threshold of <20% may simply not be achievable for lower-expressing genes even when the actual standard deviation is low.

Supplementary Table 1: Burden of CDx indications in oncology patients

CDx Biomarker	Prevalence reported in literature	Prevalence in Caris Life Science repository, %
***PIK3CA* Alterations in BC**	~40% in advanced HR-positive, HER2-negative BC [1, 2]	36.4%
***KRAS/NRAS* Wild-type in CRC**	~50% [3, 4]	51.2%
***BRAF*^{V600E} Mutations in CRC**	5–12% [5–7]	7.9%
***BRAF*^{V600E} or *BRAF*^{V600K} Mutations in Melanoma**	26–40% and 3–15% for *BRAF* V600E and V600K, respectively [8–10]	23.8% for *BRAF* V600E and 8.2% for V600K
***EGFR* exon19 Deletions or L858R Mutations in NSCLC**	7–10% [11]	L858R 4.2%; exon 19 deletions 5.7%
**Microsatellite Instability (MSI) in EC[a] and Solid Tumors**	10–19%; 26% EC [12]	3.5% solid tumors; 31.2% EC

[a]CDx indication is for microsatellite stable (MSS)/mismatch repair proficient (MMRp) (not MSI-high) EC. Abbreviations: BC: breast carcinoma; CRC: colorectal adenocarcinoma; EC: endometrial carcinoma; HR: hormone receptor; MSI: microsatellite instability; NSCLC: non-small cell lung cancer.

REFERENCES (SUPPLEMENTARY TABLE 1)

1. André F, Ciruelos E, Rubovszky G, Campone M, Loibl S, Rugo HS, Iwata H, Conte P, Mayer IA, Kaufman B, Yamashita T, Lu YS, Inoue K, et al, and SOLAR-1 Study Group. Alpelisib for *PIK3CA*-Mutated, Hormone Receptor-Positive Advanced Breast Cancer. N Engl J Med. 2019; 380:1929–40. https://doi.org/10.1056/NEJMoa1813904. [PubMed]
2. Martínez-Sáez O, Chic N, Pascual T, Adamo B, Vidal M, González-Farré B, Sanfeliu E, Schettini F, Conte B, Brasó-Maristany F, Rodríguez A, Martínez D, Galván P, et al. Frequency and spectrum of PIK3CA somatic mutations in breast cancer. Breast Cancer Res. 2020; 22:45. https://doi.org/10.1186/s13058-020-01284-9. [PubMed]
3. Sorich MJ, Wiese MD, Rowland A, Kichenadasse G, McKinnon RA, Karapetis CS. Extended RAS mutations and anti-EGFR monoclonal antibody survival benefit in metastatic colorectal cancer: a meta-analysis of randomized, controlled trials. Ann Oncol. 2015; 26:13–21. https://doi.org/10.1093/annonc/mdu378. [PubMed]
4. Udar N, Iyer A, Porter M, Haigis R, Smith S, Dhillon S, Meier K, Ward D, Lu J, Wenz P, Buchner L, Dunn T, Wise A, et al. Development and Analytical Validation of a DNA Dual-Strand Approach for the US Food and Drug Administration-Approved Next-Generation Sequencing-Based Praxis Extended RAS Panel for Metastatic Colorectal Cancer Samples. J Mol Diagn. 2020; 22:159–78. https://doi.org/10.1016/j.jmoldx.2019.09.009. [PubMed]
5. Davies MA, Saiag P, Robert C, Grob JJ, Flaherty KT, Arance A, Chiarion-Sileni V, Thomas L, Lesimple T, Mortier L, Moschos SJ, Hogg D, Márquez-Rodas I, et al. Dabrafenib plus trametinib in patients with BRAF^{V600}-mutant melanoma brain metastases (COMBI-MB): a multicentre, multicohort, open-label, phase 2 trial. Lancet Oncol. 2017; 18:863–73. https://doi.org/10.1016/S1470-2045(17)30429-1. [PubMed]
6. Venderbosch S, Nagtegaal ID, Maughan TS, Smith CG, Cheadle JP, Fisher D, Kaplan R, Quirke P, Seymour MT, Richman SD, Meijer GA, Ylstra B, Heideman DA, et al. Mismatch repair status and BRAF mutation status in metastatic colorectal cancer patients: a pooled analysis of the CAIRO, CAIRO2, COIN, and FOCUS studies. Clin Cancer Res. 2014; 20:5322–30. https://doi.org/10.1158/1078-0432.CCR-14-0332. [PubMed]
7. Tran B, Kopetz S, Tie J, Gibbs P, Jiang ZQ, Lieu CH, Agarwal A, Maru DM, Sieber O, Desai J. Impact of BRAF mutation and microsatellite instability on the pattern of metastatic spread and prognosis in metastatic colorectal cancer. Cancer. 2011; 117:4623–32. https://doi.org/10.1002/cncr.26086. [PubMed]
8. Nepote A, Avallone G, Ribero S, Cavallo F, Roccuzzo G, Mastorino L, Conforti C, Paruzzo L, Poletto S, Carnevale Schianca F, Quaglino P, Aglietta M. Current Controversies and Challenges on BRAF V600K-Mutant Cutaneous Melanoma. J Clin Med. 2022; 11:828. https://doi.org/10.3390/jcm11030828. [PubMed]
9. Hayward NK, Wilmott JS, Waddell N, Johansson PA, Field MA, Nones K, Patch AM, Kakavand H, Alexandrov LB, Burke H, Jakrot V, Kazakoff S, Holmes O, et al. Whole-genome landscapes of major melanoma subtypes. Nature. 2017; 545:175–80. https://doi.org/10.1038/nature22071. [PubMed]

10. Cancer Genome Atlas Network. Genomic Classification of Cutaneous Melanoma. Cell. 2015; 161:1681–96. https://doi.org/10.1016/j.cell.2015.05.044. [PubMed]

11. Graham RP, Treece AL, Lindeman NI, Vasalos P, Shan M, Jennings LJ, Rimm DL. Worldwide Frequency of Commonly Detected EGFR Mutations. Arch Pathol Lab Med. 2018; 142:163–67. https://doi.org/10.5858/arpa.2016-0579-CP. [PubMed]

12. Lorenzi M, Amonkar M, Zhang J, Mehta S, Liaw KL. Epidemiology of Microsatellite Instability High (MSI-H) and Deficient Mismatch Repair (dMMR) in Solid Tumors: A Structured Literature Review. Journal of Oncology 2020; 2020:1807929. https://doi.org/10.1155/2020/1807929.

Supplementary Table 2: Comparator assays for MI Cancer Seek validation

Test	Comparator assay	Testing laboratory
***PIK3CA* Alterations in BC**	Qiagen *therascreen PIK3CA* RGQ PCR Kit (PMA: P190004)	Quest Diagnostics | med fusion
***KRAS/NRAS* Wild-type in CRC**	Praxis Extended RAS Panel (PMA: P160038)	Quest Diagnostics Nichols Institute
***BRAF*^{V600E} Mutations in CRC**	Qiagen *therascreen* BRAF V600E RGQ PCR Kit (PMA: P190026)	Quest Diagnostics | med fusion
***BRAF*^{V600E} or *BRAF*^{V600K} Mutations in Melanoma**	bioMérieux’s THxID BRAF Kit (PMA: P120014)	Quest Diagnostics Nichols Institute
***EGFR* exon19 Deletions or L858R Mutations in NSCLC**	Roche cobas *EGFR* Mutation Test V2 (PMA: P120019)	Quest Diagnostics Nichols Institute
**Microsatellite Instability (MSI) in EC and Solid Tumors**	Ventana MMR RxDx Panel (PMA: P200019)	Caris Life Sciences
**SNV, INDEL**	PGDx elio™ tissue complete (510(K): K192063)	Caris Life Sciences[a]
***ERBB2* CNA**	PathVysion HER2 DNA Probe Kit (PMA:P980024)	PhenoPath Laboratories (A Quest Diagnostic Company)
**TMB**	External WES assay	Personalis, Inc.

[a]Caris operators were trained by PGDx personnel to execute the assay. Abbreviations: BC: breast carcinoma; CNA: copy number amplification; CRC: colorectal carcinoma; EC: endometrial carcinoma; INDEL: insertion/deletion; MSI: microsatellite instability; NSCLC: non-small cell lung cancer; SNV: single nucleotide variant; TMB: tumor mutational burden; WES: whole exome sequencing.

Supplementary Table 3: Concordance table with CCD1, CCD2 and MI Cancer Seek (FCD) results with all eligible samples, excluding invalids

		CCD1+/CCD2+	CCD1+/CCD2−	CCD1−/CCD2+	CCD1−/CCD2-
**MSI (Solid Tumors)**	**FCD+**	193	2	0	3
	**FCD−**	5	1	3	194
	**Total**	198	3	3	197
**MSI (EC)**	**FCD+**	120	1	0	3
	**FCD−**	2	0	2	123
	**Total**	122	1	2	126
***BRAF*$^{V600E/K}$ (Melanoma)**	**FCD+**	173	0	0	1
	**FCD−**	2	2	0	152
	**Total**	175	2	0	153

***BRAF*^{V600E} (CRC)**	**FCD+**	176	1	0	0
	**FCD−**	1	0	0	174
	**Total**	177	1	0	174
***KRAS/NRAS* Wild-type (CRC)**	**FCD+**	118	0	0	4
	**FCD−**	0	1	0	139
	**Total**	118	1	0	143
***EGFR* exon 19 Deletions or L858R Mutations (NSCLC)**	**FCD+**	151	0	0	1
	**FCD−**	3	0	0	160
	**Total**	154	0	0	161
***PIK3CA* Alterations (BC)**	**FCD+**	174	0	0	0
	**FCD−**	1	2	1	165
	**Total**	175	2	1	166

Abbreviations: BC: breast carcinoma; CCD: comparator companion diagnostic; CI: confidence interval; CRC: colorectal carcinoma; EC: endometrial carcinoma; FCD: follow-on companion diagnostic; MSI: microsatellite instability; NPA: negative percent agreement; NSCLC: non-small cell lung cancer; PPA: positive percent agreement.

Supplementary Table 4: Conditional agreements, excluding invalids

	**MSI (Solid Tumors)**		***EGFR* exon 19 Deletions or L858R Mutations (NSCLC)**	
	**Unadjusted for Prevalence**	**Adjusted for Prevalence (3.6%)**	**Unadjusted for Prevalence**	**Adjusted for Prevalence (10%)**
$\mathbf{PPA_{C1C2}}$	98.5%	98.5%	100%	100%
$\mathbf{PPA_{C1F}}$	97.0%	97.0%	98.1%	98.1%
$\mathbf{PPA_{C2C1}}$	98.5%	71.0%	100%	100%
$\mathbf{PPA_{C2F}}$	96.0%	69.2%	98.1%	98.1%
$\mathbf{NPA_{C1C2}}$	98.5%	98.5%	100%	100%
$\mathbf{NPA_{C1F}}$	98.5%	98.5%	99.4%	99.9%
$\mathbf{NPA_{C2C1}}$	98.5%	99.9%	100%	100%
$\mathbf{NPA_{C2F}}$	97.5%	98.4%	99.4%	99.9%
	**MSI (EC)**		***PIK3CA* (BC)**	
	**Unadjusted for Prevalence**	**Adjusted for Prevalence (31.2%)**	**Unadjusted for Prevalence**	**Adjusted for Prevalence (36.4%)**
$\mathbf{PPA_{C1C2}}$	99.2%	99.2%	98.9%	97.9%
$\mathbf{PPA_{C1F}}$	98.4%	98.4%	98.3%	96.9%
$\mathbf{PPA_{C2C1}}$	98.4%	96.6%	99.4%	98.9%
$\mathbf{PPA_{C2F}}$	96.8%	95.1%	98.9%	97.9%
$\mathbf{NPA_{C1C2}}$	98.4%	98.4%	99.4%	99.4%
$\mathbf{NPA_{C1F}}$	97.7%	97.7%	100.0%	100%
$\mathbf{NPA_{C2C1}}$	99.2%	99.6%	98.8%	98.8%
$\mathbf{NPA_{C2F}}$	96.9%	97.3%	100.0%	100%

	BRAF$^{V600E/K}$ (Melanoma)		*KRAS/NRAS* Wild-type (CRC)	
	**Unadjusted for Prevalence**	**Adjusted for Prevalence (31%)**	**Unadjusted for Prevalence**	**N/A**
**PPA_{C1C2}**	98.9%	97.2%	99.2%	N/A
**PPA_{C1F}**	97.7%	95.3%	99.2%	N/A
**PPA_{C2C1}**	100%	100%	100%	N/A
**PPA_{C2F}**	98.9%	98.0%	100%	N/A
**NPA_{C1C2}**	100%	100%	100%	N/A
**NPA_{C1F}**	99.3%	99.7%	97.2%	N/A
**NPA_{C2C1}**	98.7%	98.7%	99.3%	N/A
**NPA_{C2F}**	99.4%	99.7%	97.2%	N/A
	BRAF^{V600E} (CRC)			
	**Unadjusted for Prevalence**	**Adjusted for Prevalence (10%)**		
**PPA_{C1C2}**	99.4%	99.5%		
**PPA_{C1F}**	99.4%	95.1%		
**PPA_{C2C1}**	100%	100%		
**PPA_{C2F}**	99.4%	95.1%		
**NPA_{C1C2}**	100%	100%		
**NPA_{C1F}**	100%	100%		
**NPA_{C2C1}**	99.4%	99.9%		
**NPA_{C2F}**	99.4%	99.9%		

Abbreviations: BC: breast carcinoma; CDx: companion diagnostic; CI: confidence interval; CRC: colorectal carcinoma; EC: endometrial carcinoma; MSI: microsatellite instability; NPA: negative percent agreement; NSCLC: non-small cell lung cancer; PPA: positive percent agreement.

Supplementary Table 5: Concordance analysis for non-inferiority hypothesis tests, excluding invalids

**MSI (Solid Tumors)**	**Point estimate (ε)**	**95% two-sided CI (ε1, ε0)**
	**Prevalence (3.6%)**	
**ζPPA1**	1.5	(−1.0, 4.0)
**ζPPA2**	1.8	(0.4, 3.7)
**ζNPA1**	0	(−2.5, 2.5)
**ζNPA2**	15	(0.0, 3.5)
**MSI (EC)**	**Prevalence (31.2%)**	
**ζPPA1**	0.8	(−1.6, 3.3)
**ζPPA2**	1.5	(0.0, 3.9)
**ζNPA1**	0.8	(−2.3, 3.9)
**ζNPA2**	2.4	(0.0, 5.5)

BRAF$^{V600E/K}$ **(Melanoma)**		**Prevalence (31%)**
**ζPPA1**	1.9	(0.0, 5.2)
**ζPPA2**	2.0	(0.0, 5.3)
**ζNPA1**	0.3	(0.0, 0.8)
**ζNPA2**	−1.0	(−3.1, 0.5)
BRAF^{V600E} **(CRC)**		**Prevalence (10%)**
**ζPPA1**	4.4	(−1.7, 13.2)
**ζPPA2**	4.9	(0.0, 13.4)
**ζNPA1**	0.0	(0.0, 0.0)
**ζNPA2**	0.0	(0.0, 0.0)
KRAS/NRAS **Wild-type (CRC)**		**Unadjusted for prevalence**
**ζPPA1**	0.0	(−3.5, 3.5)
**ζPPA2**	0.0	(−3.2, 3.2)
**ζNPA1**	2.8	(−0.3, 6.9)
**ζNPA2**	2.1	(−1.5, 6.3)
EGFR **exon 19 Deletions or L858R Mutations (NSCLC)**		**Prevalence (10%)**
**ζPPA1**	1.9	(0.0, 4.5)
**ζPPA2**	1.9	(0.0, 4.5)
**ζNPA1**	0.1	(0.0, 0.2)
**ζNPA2**	0.1	(0.0, 0.2)
PIK3CA **Alterations (BC)**		**Prevalence (36.4%)**
**ζPPA1**	1.0	(0.0, 3.9)
**ζPPA2**	1.1	(0.0, 3.9)
**ζNPA1**	−0.6	(−1.9, 0.0)
**ζNPA2**	−1.2	(−3.0, 0.0)

The primary concordance analysis was performed excluding samples with an invalid result on either CCD1, CCD2, or FCD. The analysis was performed at the prevalence level in the Caris repository. Two additional prevalence levels were also analyzed for each study except for *KRAS/NRAS*, which did not require prevalence adjustment. All analyses met acceptance criteria (data not shown). Abbreviations: BC: breast carcinoma; CI: confidence interval; CNA: copy number amplification; CRC: colorectal carcinoma; EC: endometrial carcinoma; MSI: microsatellite instability; NPA: negative percent agreement; NSCLC: non-small cell lung cancer; PPA: positive percent agreement.

Supplementary Table 6: Reportable gene list

ABL1	*BRCA1*	*CYLD*	*FGFR1*	*JAK2*	*MPL*	*PDGFRA*	*RAF1*	*STAG2*
ACVR1	*BRCA2*	*DDR2*	*FGFR2*	*JAK3*	*MRE11*	*PDGFRB*	*RASA1*	*STAT3*
AIP	*BRIP1*	*DICER1*	*FGFR3*	*KDM5C*	*MSH2*	*PIK3CA*	*RB1*	*STK11*
AKT1	*BTK*	*DNMT3A*	*FGFR4*	*KDM6A*	*MSH3*	*PIK3CB*	*RET*	*SUFU*
AKT2	*CALR*	*EGFR*	*FH*	*KDR*	*MSH6*	*PIK3R1*	*RHOA*	*TCF7L2*
AKT3	*CARD11*	*EP300*	*FLCN*	*KEAP1*	*MTOR*	*PIK3R2*	*RNF43*	*TERT*
ALK	*CBFB*	*EPHA2*	*FLT1*	*KIT*	*MUTYH*	*PIM1*	*ROS1*	*TET2*
AMER1	*CCND1*	*ERBB2*	*FLT3*	*KLF4*	*MYC*	*PMS2*	*RUNX1*	*TMEM127*

APC	CCND2	ERBB3	FOXA1	KMT2A	MYCN	POLD1	SDHA	TNFAIP3
AR	CCND3	ERBB4	FOXL2	KMT2C	MYD88	POLE	SDHAF2	TNFRSF14
ARAF	CD79B	ERCC2	FUBP1	KMT2D	NBN	POT1	SDHB	TP53
ARID1A	CDC73	ESR1	GATA3	KRAS	NF1	PPP2R1A	SDHC	TRAF7
ARID2	CDH1	EZH2	GNA11	LZTR1	NF2	PPP2R2A	SDHD	TSC1
ASXL1	CDK12	FANCA	GNA13	MAP2K1	NFE2L2	PRDM1	SETD2	TSC2
ATM	CDK4	FANCB	GNAQ	MAP2K2	NFKBIA	PRKACA	SF3B1	U2AF1
ATRX	CDKN1B	FANCC	GNAS	MAP2K4	NOTCH1	PRKAR1A	SMAD2	VHL
AXIN2	CDKN2A	FANCD2	H3F3A	MAP3K1	NPM1	PRKDC	SMAD4	WRN
B2M	CHEK1	FANCE	H3F3B	MAPK1	NRAS	PTCH1	SMARCA4	WT1
BAP1	CHEK2	FANCF	HIST1H3B	MAX	NSD1	PTEN	SMARCB1	XPO1
BARD1	CIC	FANCG	HNF1A	MED12	NSD2	PTPN11	SMARCE1	XRCC1
BCL2	CREBBP	FANCI	HOXB13	MEF2B	NTHL1	RAC1	SMO	
BCL9	CSF1R	FANCL	HRAS	MEN1	NTRK1	RAD50	SOCS1	
BCOR	CTCF	FANCM	IDH1	MET	NTRK2	RAD51B	SOS1	
BLM	CTNNA1	FAS	IDH2	MITF	NTRK3	RAD51C	SPEN	
BMPR1A	CTNNB1	FAT1	IRF4	MLH1	PALB2	RAD51D	SPOP	
BRAF	CXCR4	FBXW7	JAK1	MLH3	PBRM1	RAD54L	SRC	

Supplementary Table 7: Tumor content LoD for SNVs, INDELs, CDx variants, and *ERBB2* CNAs

		Mean % Tumor content[a]
**Tumor profiling**	SNV	12.1
	INDEL	9.1
	Combined SNV/INDEL	10.5
	ERBB2 CNA	15.0
**CDx variants**	SNV	12.0
	INDEL	11.9
	Combined SNV/INDEL	12.0

[a]For SNVs and INDELS: represents mean % tumor content at variant frequency LoD (this study was designed to determine variant frequency LoD so samples were diluted to achieve target variant frequencies; tumor content of each sample was examined post-hoc). For *ERBB2* CNAs: represents target % tumor content across replicates (this study was designed to determine tumor content LoD so samples were diluted to achieve target tumor content; copy number of each sample was examined post-hoc).

Supplementary Table 8: Limit of blank

Category	Per Position false positive rate	Per sample false positive rate[a]
**Level 1: *BRAF*^{V600E}**	0%	0%
**Level 1: *BRAF*^{V600K}**	0%	0%
**Level 1: *EGFR* Exon 19 deletions**	0%	0%
**Level 1: *EGFR* Exon 21 substitution mutations**	0%	0%
**Level 1: *PIK3CA* C420R, E542K, E545A, E545D [1635G>T only], E545G, E545K, Q546E, Q546R, H1047L, H1047R, H1047Y**	0%	0%

**Level 1: *KRAS* SNVs**	0%	0%
**Level 1: *NRAS* SNVs**	0%	0%
**Level 1: MSI High**	N/A	0%
**Level 2: *ERBB2* CNAs**	N/A	0%
**Level 2: Panel-wide SNVs (*n* = 924)**	0%	0%
**Level 2: Panel-wide Indels (*n* = 950)**	0%	0%
**Level 3: Panel-wide SNVs (*n* = 50710)**	0.0035% (3/50710*168)	1.8% (3/168)
**Level 3: Panel wide Indels (*n* = 115407)**	0%	0%

[a]Calculated from 2 reagent lots (*n* = 168 data points). Abbreviations: BC: breast carcinoma; CNA: copy number amplification; INDEL: insertion/deletion; MSI: microsatellite instability; MNV: multi-nucleotide variant; NSCLC: non-small cell lung cancer; SNV: single nucleotide variant; TMB: tumor mutational burden.

Supplementary Table 9: Interfering substances

	**INDEL**		**SNV**		**MSI**		***ERBB2* CNA**		**TMB**
**Interfering substance**	**PPA**	**NPA**	**PPA**	**NPA**	**PPA**	**NPA**	**PPA**	**NPA**	**Mean difference in TMB (95% CI)**
**80% Ethanol (10% by volume)**	100%	100%	100%	100%	100%	100%	100%	100%	−0.06 (−0.2, 0.1)
**Paraffin (4x expected residual-0.72 g)**	100%	100%	100%	100%	100%	100%	100%	100%	−0.1 (−0.3, −0.09)
**Xylene (6x expected residual-6mL)**	100%	100%	100%	100%	100%	100%	100%	100%	0.6 (−0.1, 0.2)
**Proteinase K (0.09 mg/mL)**	100%	100%	97.4%	100%	93.3%	100%	100%	98.7%	0.8 (−0.03, 2.0)
**Hemoglobin (4 mg/mL)**	100%	100%	100%	100%	91.3%	97.6%	100%	100%	−0.08 (−0.2, 0.06)
**Calcium/calcium phosphate (5 mmol/L)**	N/A	N/A	100%	100%	100%	93.8%[a]	N/A	N/A	0.5 (−0.2, 1.0)
**Colloid (1:2 ratio colloid to tumor tissue)**	N/A	N/A	100%	100%	50%[a]	100%	N/A	N/A	0.2 (−0.3, 0.8)
**Mucin (1 mg/mL)**	100%	100%	100%	100%	100%	100%	N/A	N/A	0.5 (−3.0, 4.0)
**Conjugated bile acids (3 umol/L)**	N/A	N/A	100%	100%	100%	100%	N/A	N/A	0.3 (−2.0, 2.0)

[a]Discordant calls due to threshold MSI status; numerical MSI frameshift counts were not statistically different between control and colloid. Abbreviations: CI: confidence interval; CNA: copy number amplification; INDEL: insertion/deletion; MSI: microsatellite instability; NPA: negative percent agreement; PPA: positive percent agreement; SNV: single nucleotide variant; TMB: tumor mutational burden.

Supplementary Table 10: Comparator assays for MI Tumor Seek Hybrid validation

LDT Capability[a]	Comparator assay(s)
ERBB2 (HER2) CNA in BC	Ventana's PATHWAY anti-HER2/neu (4B5) IHC test
CNVs (including 1p19q co-deletion)	MI Exome
7+/10- co-occurrence	aCGH Children's Hospital, Los Angeles
ALK fusion status in NSCLC	ALK (D5F3) CDx Assay
SVs/fusions	MI Transcriptome, FusionPlex, TSO500, or PGDx elio™ tissue complete
SSVs (*MET* exon 14 skipping, *AR*v7, *EGFR*vIII)	MI Transcriptome
HLA	MI Exome
LoH	MI Exome
HRD	MI Exome and<br>Myriad MyChoice
FOLFIRSTai	MI Exome
GPSai	N/A[b]
HPV	NeoGenomics PCR test
EBV	EBER ISH assay
MCPyV	MCPyV IHC assay (Mayo Clinic Labs)

[a]LDT capabilities included here exclude capabilities subsequently validated in FDA tumor profiling and CDx studies (other than *ERBB2* CNA). [b]Compared to pathologist-submitted diagnosis. Abbreviations: EBV: Epstein-Barr Virus; GPS: genomic prevalence score; HLA: human leukocyte antigen; HPV: human papilloma virus; HRD: homologous recombination deficiency; IHC: immunohistochemistry; ISH: in situ hybridization; LDT: laboratory developed test; LoH: loss of heterozygosity; MCPyV: Merkel Cell Polyomavirus.

Supplementary Table 11: Summary of PPA and NPA for MI Tumor Seek Hybrid (LDT) clinical concordance studies

Biomarker	Comparator method	Sample size *N*	PPA	NPA
**MSI status solid tumor types**	Ventana MMR RxDx Panel	46,976	94.0%<br>(93.0, 94.9)	99.9%<br>(99.9, 99.9)
**MSI status EC**	Ventana MMR RxDx Panel	3,488	96.7%<br>(95.4, 97.7)	99.6%<br>(99.3, 99.8)

Abbreviations: LDT: laboratory developed test; MSI: microsatellite instability; NPA: negative percent agreement; PPA: positive percent agreement.

Supplementary Table 12: Reportable ranges

Marker	Reported outcome	Reportable range	Included in report
**SNV, INDEL**	Detected, Not Detected, Indeterminate	for variants supported by at least five read alignments and 5% VF, detected variant is reported as pathogenic, likely pathogenic, uncertain significance, likely benign, or benign	gene, reference sequence, protein alteration, exon, DNA alteration, variant frequency; variant depth available upon request
**SVs/Fusions**	Detected, Not Detected	nReads ≥75	SV/fusion detected
**SSVs (*MET* exon 14 skipping, *AR*v7, *EGFR*vIII)**	Detected, Not Detected	*MET* exon 14 skipping: nReads ≥125<br>*AR*v7: nReads ≥100<br>*EGFR*III: nReads ≥75	SSV detected

**CNV**	Amplification detected	segment-level copies exceeding 6 with the 95% prediction interval exceeding 3.5<br>*MDM2*, *MET*: segmented copies ≥6 and unsegmented copies ≥ 4<br>*ERBB2*/HER2: ≥ 6.9 copies. *ERBB2* CNAs may also be reported if *ERBB2* has ≥4.1 copies with HER2/CEP17 ratio ≥ 2.1. HER2/CEP17 ratio is a test that measures the number of HER2 gene copies on chromosome 17 (*ERBB2*) in relation to the number of chromosome 17 centromere (CEP17) copies per nucleus.	numerical CNA value not included but available upon request; Karyotype images are also extracted from CNA data and included in report
	Amplification intermediate	segment-level copies exceeding 6 but the 95% prediction interval does not exceed 3.5<br>*ERBB2*/HER2: ≥ 3.3 copies and *ERBB2*/CEP17 ≥ 2.1. Patients with breast cancer whose samples receive *ERRB2* CNA "Intermediate" calls should be tested with another FDA approved or cleared test to ascertain *ERRB2* CNA status in their tumor.	
	Homozygous deletion	segment-level copies < 1 with the 95% confidence interval < 1 copy	
	Deletion intermediate	1 ≤ segmented copies < 1.9	
	Stable	any other copy value	
	Indeterminate	average depth < 100x	
	1p19q codeletion	both chromosome arm copy ratios < 0.8 and all copies per arm are < 2	
	7+/10- co-occurrence	>2.8 copies chr7 and <1.2 copies chr10	
**TMB**	High	≥ 10 mutations/Mb	number of mutations/Mb
	Low	< 10 mutations/Mb	
	Indeterminate	average depth < 100x	
**MSI**	High	≥ 39 frameshift mutations	number of altered MSI loci are not included
	Stable	< 39 frameshift mutations	
	Indeterminate	average depth < 100x	
**LoH**	High	≥ 16% genomic LoH	percent genomic LoH
	Equivocal	11% ≤ genomic LoH ≤ 15%	
	Low	genomic LoH < 11%%	
	Indeterminate	SNPs ≤ 3000 or sample depth < 200x	
**HLA**	Genotypes	N/A	optimal HLA genotype for the HLA-A, HLA-B and HLA-C genes
**HRD**	Positive	*BRCA1/2*-positive and/or GSS high (≥46)	reported only for ovarian cancer
	Negative	does not meet criteria for positive	
**Gene Expression**	(+) or (-) for *ERBB2* (HER2), *ER*, *PR*, and *AR*	measurable values for TPM range from 0.01 to 1,000,000	markers displayed will be determined on a lineage basis and will be reported with TPM and the accompanying percentile in the cancer type
**HPV**	Positive	≥ 300 reads	Reported only if positive for one of five subtypes
	Negative	< 300 reads	

**EBV**	Positive	≥ 100,000 reads	Reported if positive
	Negative	< 100,000 reads	
**MCPyV**	Positive	≥ 1,000 reads	Reported if positive
	Negative	< 1,000 reads	

Abbreviations: CNV: copy number variation; EBV: Epstein-Barr Virus; GSS: genomic scar score; HLA: human leukocyte antigen; HRD: homologous recombination deficiency; INDEL: insertion/deletion; LoH: loss of heterozygosity; MSI: microsatellite instability; MCPyV: Merkel Cell Polyomavirus; SNV: single nucleotide variant; SSV: splice site variant; SV: structural variant; TMB: tumor mutational burden.

Supplementary Table 13: Summary of clinical and analytical study designs

Study	Goal	Sample #	# of replicates	Conditions notes	Lineages
**Accuracy (CDx)**	Orthogonal testing against FDA-approved comparator assays.	2063[a]	1 replicates for each CCD test; 1 replicate for FCD testing	50–220 ng	Melanoma, CRC, NSCLC, BC, EC, Solid Tumors
**Accuracy (Tumor profiling)**	Orthogonal testing against validated comparator assays	500[a] (TMB, SNV, INDEL) 288[a] (*ERBB2* CNA)	1 replicate for MI Cancer Seek and 1 replicate for comparator test	50 ng	38 tumor lineages (various)
**Accuracy (LDT)**	Orthogonal testing against validated in-house or external comparator assays.	2955[b] Additional studies: 60,157[c]	1 replicate for MI Tumor Seek Hybrid and 1 replicate for comparator test	110/220 ng	36 tumor lineages
**Limit of detection[d]**	Identify the lowest variant frequency (VF) and tumor content (TC) at which 95% of test replicates produce a positive result.	VF: 29 marker positive samples (12 for CDx biomarkers); TC: 12 marker positive samples	20 replicates per dilution level (10 replicates per reagent lot)	50 ng; VF: targeted levels were 11%, 7%, 5%, 3% 1%; TC: 5 dilutions with TC varying from 5–40%	BC, CRC, Glioblastoma, Gastric Adenocarcinoma, Kidney Cancer, Low Grade Glioma, NSCLC, Melanoma, Ovarian Surface Epithelial Carcinomas, Pancreatic Adenocarcinoma, Prostatic Adenocarcinoma, EC
**Limit of blank**	Verify the ability of the assay to correctly identify non-pathogenic genotype and measure the false positive rate (or error rate).	28 wild-type (marker negative) samples	6	Normal tissue or tumor adjacent was tested	Melanoma, Extrahepatic Bile Duct Adenocarcinoma, NSCLC, Esophageal Carcinoma, CRC, Urothelial Carcinoma, Uterine Serous Carcinoma, Ovarian Surface Epithelial Carcinomas, Kidney Cancer, Thyroid Carcinoma, Liver Hepatocellular Carcinoma, Gastric Adenocarcinoma, Small Intestinal Malignancies, Pancreatic Adenocarcinoma, BC
**DNA input study[d]**	Identify the minimum input of DNA at which the assay performs consistently.	16	3-6 for each input level	1-3x LoD; Six DNA input levels tested: 25, 37.5, 50, 220, 275, 330 ng	CRC, Melanoma, BC, NSCLC, Small Intestinal Malignancies, Urothelial Carcinoma, EC, Female Genital Tract Malignancy
**Precision[d]**	Demonstrate consistency of performance between multiple parameters.	49	36 for each dilution level	1-1.5x LoD and 2-3x LoD; 3 operator teams, 3 instrument sets, 3 reagent lots across 3 non-consecutive days over a 20-day span	CRC, Melanoma, BC, NSCLC, Small Intestinal Malignancies, Prostatic Adenocarcinoma, Glioblastoma, Uterine Serous Carcinoma, Gastrointestinal Stromal Tumors (GIST), EC, Ovarian Surface Epithelial Carcinomas, Female Genital Tract Malignancy, Salivary Gland Tumors, Soft Tissue Tumors

**Interfering substances**	To identify any effect of interfering substances on test performance.	Exogenous: 17<br>Endogenous: 23	5 replicates per condition, including control (no substances spiked in)	2-3x LoD;<br>Exogenous: paraffin, xylene, Proteinase K, and 80% ethanol;<br>Endogenous: hemoglobin, colloid, calcium/calcium phosphate, mucin, and conjugated bile acids	BC, Cholangiocarcinoma, CRC, Liver Hepatocellular Carcinoma, NSCLC, Melanoma, Ovarian Surface Epithelial Carcinomas, Pancreatic Adenocarcinoma, Prostatic Adenocarcinoma, Small Intestinal Malignancies, Thyroid Carcinoma, EC
**Carryover (run to run) and cross-contamination (within run)**	To demonstrate cleanliness of the library prep and NGS workflows and no data contribution from previous runs.	30 (14 positive, 16 negative)	Varied (172 data points total)	220ng	BC, CRC, NSCLC, Melanoma, Prostatic Adenocarcinoma

[a]Indicates number of unique enrolled samples, including cases that were excluded due to poor sample quality or invalid testing results on either CCD or FCD. [b]Indicates sum of sample numbers tested for each capability. Does not indicate total unique cases, as some samples were used for validation of more than one LDT capability. [c]Additional separate studies were performed for MSI (n = 46,976), *ALK* (n = 9,213), and *ERBB2* (n = 3,968). [d]These studies used samples diluted with lineage matched cancer or normal samples. Abbreviations: CCD: comparator companion diagnostic; BC: breast carcinoma; CRC: colorectal carcinoma; EC: endometrial carcinoma; FCD: follow-on companion diagnostic; NSCLC: non-small cell lung cancer.
